# Myoscape controls cardiac calcium cycling and contractility via regulation of L-type calcium channel surface expression

**DOI:** 10.1038/ncomms11317

**Published:** 2016-04-28

**Authors:** Matthias Eden, Benjamin Meder, Mirko Völkers, Montatip Poomvanicha, Katrin Domes, M. Branchereau, P. Marck, Rainer Will, Alexander Bernt, Ashraf Rangrez, Matthias Busch, German Mouse Clinic Consortium, Thure Adler, Dirk H. Busch, Juan Antonio Aguilar-Pimentel, Markus Ollert, Alexander Götz, Holger Schulz, Cornelia Prehn, Jerzy Adamski, Lore Becker, Thomas Klopstock, Marion Horsch, Johannes Beckers, Anja Schrewe, Raffi Bekeredjian, Hugo Katus, Lillian Garrett, Sabine M. Hölter, Wolfgang Wurst, Oliver Puk, Jochen Graw, Wolfgang Hans, Jan Rozman, Martin Klingenspor, Frauke Neff, Monica Tost, Julia Calzada-Wack, Tanja Klein-Rodewald, Ildikó Rácz, Andreas Zimmer, Birgit Rathkolb, Eckhard Wolf, Christoph Lengger, Holger Maier, Claudia Stoeger, Stefanie Leuchtenberger, Valéri Gailus-Durner, Helmut Fuchs, Martin Hrabě de Angelis, Christophe Heymes, Wolfgang Rottbauer, Patrick Most, Franz Hofmann, Norbert Frey

**Affiliations:** 1 Department of Internal Medicine III (Cardiology and Angiology), University Hospital Schleswig-Holstein, Campus Kiel, 24105 Kiel, Germany; 2 German Centre for Cardiovascular Research, partner site 20246 Hamburg/24105 Kiel/23562 Lübeck, Germany; 3 Department of Internal Medicine III, University of Heidelberg, 69120 Heidelberg, Germany; 4 Department of Pharmacology, University of Technology Munich, 80333 Munich, Germany; 5 Inserm U1048 - Institut des Maladies Métaboliques et Cardiovasculaires (I2MC)/Equipe 13, Bat L4 1er étage C.H.U Rangueil 1 av Jean Poulhès - BP 84225 31432 Toulouse Cedex 4, France; 6 German Mouse Clinic, Institute of Experimental Genetics, Helmholtz Zentrum München, German Research Center for Environmental Health, 85764 Neuherberg, Germany; 7 Department of Internal Medicine III, Cardiology and Angiology, University Hospital of Ulm, 89081 Ulm, Germany; 8 German Centre for Cardiovascular Research, partner site 69120 Heidelberg/68167 Mannheim, Germany; 9 German Mouse Clinic, Institute of Experimental Genetics, Helmholtz Zentrum München, German Research Center for Environmental Health GmbH, Ingolstaedter Landstrasse 1, 85764 Neuherberg, Germany; 10 Institute for Medical Microbiology, Immunology and Hygiene, Technical University of Munich, Trogerstrasse 9, 81675 Munich, Germany; 11 Department of Dermatology and Allergy, Biederstein, Klinikum rechts der Isar, Technische Universität München (TUM), Biedersteiner Str. 29, 80802 Munich, Germany; 12 Comprehensive Pneumology Center, Institute of Lung Biology and Disease, Helmholtz Zentrum München, German Research Center for Environmental Health (GmbH), Ingolstädter Landstraße 1, 85764 Neuherberg, Germany; 13 Department of Neurology, Friedrich-Baur-Institut, Ludwig-Maximilians-Universität München, Ziemssenstrasse 1a, 80336 Munich, Germany; 14 Institute of Pathology, Helmholtz Zentrum München, German Research Center for Environmental Health GmbH, Ingolstaedter Landstrasse 1, 85764 Neuherberg, Germany; 15 Institute of Developmental Genetics, Helmholtz Zentrum München, German Research Center for Environmental Health GmbH, Ingolstaedter Landstrasse 1, 85764 Neuherberg, Germany; 16 Molecular Nutritional Medicine, Technische Universität München, Else Kröner-Fresenius Center for Nutritional Medicine, 85350 Freising, Germany; 17 ZIEL–Center for Nutrition and Food Sciences, Technische Universität München, 85350 Freising, Germany; 18 Clinical Research Group Molecular Allergology, Center of Allergy and Environment Munich (ZAUM), Technische Universität München (TUM), and Institute for Allergy Research, Helmholtz Zentrum München, German Research Center for Environmental Health, 85764 Neuherberg, Germany; 19 Institute of Molecular Psychiatry, University of Bonn, Sigmund-Freud-Strasse 25, 53127 Bonn, Germany; 20 Ludwig-Maximilians-Universität München, Gene Center, Institute of Molecular Animal Breeding and Biotechnology, Feodor-Lynen Strasse 25, 81377 Munich, Germany; 21 Developmental Genetics, Center of Life and Food Sciences Weihenstephan, Technische Universität München, Ingolstaedter Landstrasse 1, 85764 Neuherberg, Germany; 22 Max Planck Institute of Psychiatry, Kraepelinstr. 2-10, 80804 Munich, Germany; 23 Deutsches Institut für Neurodegenerative Erkrankungen (DZNE) Site Munich, Schillerstrasse 44, 80336 Munich, Germany; 24 Munich Cluster for Systems Neurology (SyNergy), Adolf-Butenandt-Institut, Ludwig-Maximilians-Universität München, Schillerstrasse 44, 80336 Munich, Germany; 25 Experimental Genetics, Center of Life and Food Sciences Weihenstephan, Technische Universität München, Ingolstaedter Landstrasse 1, 85764 Neuherberg, Germany; 26 German Center for Diabetes Research (DZD), Ingolstaedter Landstraße 1, 85764 Neuherberg, Germany; 27 German Network for Mitochondrial Disorders (mitoNET), Germany; 28 German Center for Vertigo and Balance Disorders, 81377 Munich, Germany

## Abstract

Calcium signalling plays a critical role in the pathogenesis of heart failure. Here we describe a cardiac protein named Myoscape/FAM40B/STRIP2, which directly interacts with the L-type calcium channel. Knockdown of Myoscape in cardiomyocytes decreases calcium transients associated with smaller Ca^2+^ amplitudes and a lower diastolic Ca^2+^ content. Likewise, L-type calcium channel currents are significantly diminished on Myoscape ablation, and downregulation of Myoscape significantly reduces contractility of cardiomyocytes. Conversely, overexpression of Myoscape increases global Ca^2+^ transients and enhances L-type Ca^2+^ channel currents, and is sufficient to restore decreased currents in failing cardiomyocytes. *In vivo*, both Myoscape-depleted morphant zebrafish and Myoscape knockout (KO) mice display impairment of cardiac function progressing to advanced heart failure. Mechanistically, Myoscape-deficient mice show reduced L-type Ca^2+^currents, cell capacity and calcium current densities as a result of diminished LTCC surface expression. Finally, Myoscape expression is reduced in hearts from patients suffering of terminal heart failure, implying a role in human disease.

## Abstract (web summary)

Heart failure is a major public health issue but due to our poor disease understanding the current therapies are symptomatic. Here the authors identify Myoscape as a novel cardiac protein regulating membrane localization of the L-type calcium channel and heart's contractile force, thus promising new therapeutic avenues for heart failure.

## Graphical abstract

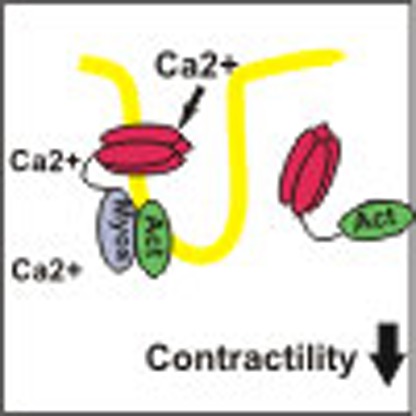

The L-type Ca^2+^channel (LTCC) enables action potential-driven calcium influx into a variety of excitable cells^1^. The regulation of calcium fluxes occurs at the level of single channel activity and precise channel sublocalization within the plasma membrane^2^. In cardiomyocytes, this microdomain-confined calcium influx controls the action potential duration and initiates calcium-induced calcium release and subsequent heart muscle contraction^3^,^4^. Moreover, it also affects intracellular signalling pathways and gene regulation^4^,^5^,^6^. In specific membrane invaginations termed t-tubules, every LTCC faces several ryanodine receptors on the sarcoplasmic reticulum to form a couplon^7^. Changes in t-tubule density, length, width and in the composition of couplon-associated proteins occur in various disease states, such as cardiac hypertrophy and heart failure^8^. Targeting of LTCCs to t-tubules is believed to control contractility via calcium-induced calcium release, while caveolae-associated LTCCs are thought to be involved in signalling, for example, regulation of cardiomyocyte hypertrophy^3^,^9^,^10^.

Modulation of the LTCC pore opening predominantly occurs at its cytosolic C-terminal region, which contains protein–protein interaction sites that can affect either function or localization^11^,^12^,^13^,^14^. Specifically, it contains various phosphorylation residues that allow fast regulatory responses^13^, as well as the IQ motif, which is a specific interaction domain for calmodulin^15^. Calmodulin represents the major calcium-dependent modulator of LTCC function, promoting either calcium-dependent inactivation (CDI) or calcium-dependent facilitation (CDF)^16^,^17^,^18^. Knock-in mice expressing LTCCs with truncated C termini show reduced LTCC surface density and impaired Ca^2+^ currents^19^.

In non-cardiac cells, endoplasmatic reticulum (ER) retention, lateral diffusion and internalization of LTCCs in cytosolic vesicles represent an important additional regulatory mechanism of calcium cycling^2^,^20^. However, LTCC surface delocalization or internalization in cardiomyocytes is still poorly understood. It has been shown that membrane-bound LTCCs have a much shorter half-life (3 h) compared with cellular LTCCs (more than 25 h)^21^,^22^. Thus, it seems conceivable that alterations in these mechanisms would result in impaired LTCC activity and/or subcellular localization, ultimately leading to cardiac calcium mishandling, contractile deficits and heart failure^3^.

In searching for novel candidate genes involved in the pathophysiology of heart failure, we identified a 834 amino acid protein, FAM40B/KIAA1170/Strip2, which we termed Myoscape (myocardium-expressed, calcium channel-associated protein). Myoscape directly binds to the C-terminal tail domain of the LTCC. Knockdown of Myoscape in cardiomyocytes results in the reduction of LTCC surface expression and the reduction of calcium channel currents. Consistently, Myoscape ablation in cardiomyocytes *in vitro* or in zebrafish and in genetically engineered mice *in vivo* results in reduced contractility and progressive heart failure.

## Results

### Myoscape localizes to sarcolemmal t-tubules

On the basis of the notion that proteins specifically expressed in the heart are likely to play a role in cardiac pathophysiology^23^, we systematically screened the expressed sequence tag (EST) databases for sequences predominantly found in cardiac complementary DNA (cDNA) libraries^24^. Data extraction relied on the T-STAG (Tissue-Specific Transcripts and Genes; http://tstag.molgen.mpg.de/) and the Unigene (National Center for Biotechnology; http://www.ncbi.nlm.nih.gov/unigene) databases.

**Figure 1:**
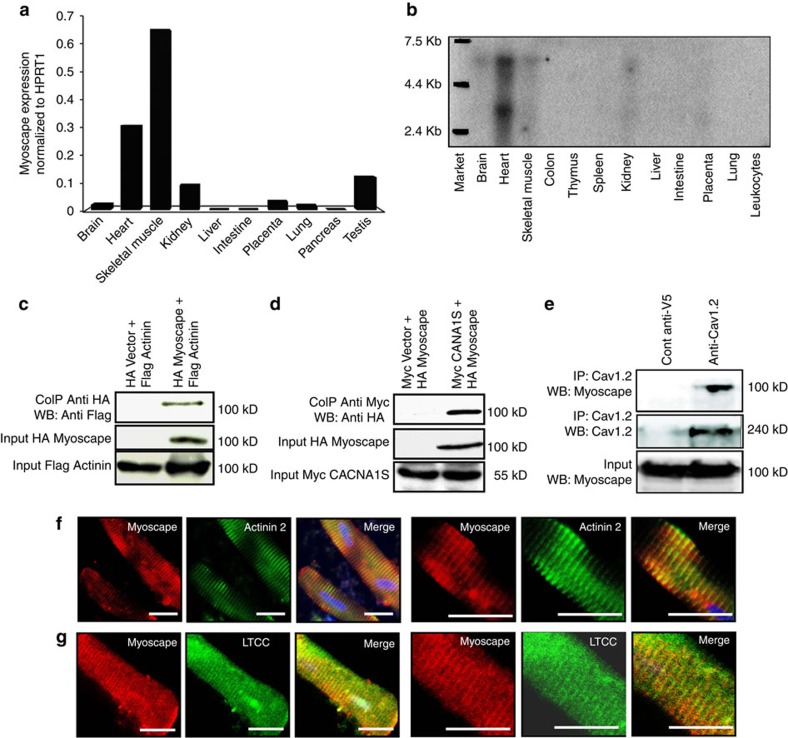
Myoscape expression profile and subcellular localization. (**a**) Mouse muscle and heart *Myoscape* mRNA expression profile confirmed by HPRT1-normalized quantitative qPCR and (**b**) additional Northern blot analyses of multiple human tissues using Myoscape-specific probes and primers. Confirmation of the interaction between α-actinin 2 (**c**) and the skeletal muscle-specific pore-forming unit (**d**) of the LTCC (CACNA 1S) with myc-tagged Myoscape by co-immunoprecipitation of the proteins in HEK-T cells. (**e**) Immunoprecipitation performed with endogenous proteins isolated from mouse heart using anti-V5 (control) or anti-Cav1.2 antibody followed by immunoblotting performed with Myoscape antibody, indicates the physiological interaction between Cav1.2 and Myoscape. (**f**–**g**) Confocal Immunofluorescence analysis of Myoscape expression in isolated adult rat ventricular myocytes shows a striated signal. Myoscape is localized to couplons near the sarcomeric z-band/t-tubule interface, as evidenced by coimmunostaining of Myoscape and actinin 2 and LTCC and RYR2. Scale bars, 20 μm.

Several of the identified ESTs corresponded to the human Unigene cluster Hs.489988 or the *Mus musculus* Unigene cluster Mm.56097. ESTs in these clusters were significantly enriched in the heart compared with other tissues (Supplementary Fig. 1a) and are predicted to encode an open reading frame (ORF) termed *FAM40B* or *STRIP2* (ref. 25). Molecular cloning of human and murine *STRIP2/FAM40B/Myoscape* complete ORFs confirmed the predicted amino acid sequences (NP_001127808.1 and NP_796178.2). To confirm Myoscape anticipated expression pattern in human tissues, we performed quantitative real-time PCR experiments (Fig. 1a). Similar to rodents, a strong expression in human heart and skeletal muscle was observed, while other tissues revealed only a weak signal. Hybridization of northern blots with either human (Fig. 1b) or murine (Supplementary Fig. 1c) Myoscape-specific cDNA probes confirmed heart and skeletal muscle gene expression. HPRT1 served as normalization control in these samples and showed similar expression levels in these different tissues (Supplementary Fig. 1b).

Given Myoscape's abundance in myocardial tissue, we next aimed to determine its protein expression pattern and its subcellular localization. A western blot of human tissues confirmed the protein expression of Myoscape protein in human heart and skeletal muscle, as well as relevant expression in brain and kidney, at the predicted size of ∼105 kDa (Supplementary Fig. 1d). Analysis of cellular subfractions revealed that Myoscape was equally detectable in cytosolic and membrane fractions of rat heart extracts (Supplementary Fig. 1e). Protein sequence alignments between human (NM_020704.2), mouse (NM_177204.3), rat and zebrafish gene homologues revealed high evolutionary conservation (Supplementary Fig. 1f).

### Myoscape interacts with α-actinin 2 and the LTCC

To identify protein interaction partners for Myoscape, we performed yeast two-hybrid experiments and screened human heart and skeletal muscle-derived cDNA libraries. We found several clones encoding for α-actinin 2 and the skeletal muscle isoform of the LTCC pore-forming unit (Cav1.1/CACNA1S). Co-immunoprecipitation experiments confirmed these interactions (Fig. 1c,d). We also confirmed the interaction of Myoscape with the cardiac specific isoform of the endogenous LTCC (*CAV1.2/CACNA1C*) in mouse heart lysate (Fig. 1e) and in neonatal rat ventricular cardiomyocytes (NRVCM), utilizing an adenovirus encoding full-length Myoscape (Supplementary Fig. 3b). The interaction domains were mapped by comparison of the sequences of the different positive Y2H clones. Moreover, we could confirm the predicted interaction site in yeast by performing additional glutathione-S-transferase (GST) pull-down experiments with either full-length LTCC or the predicted LTCC interaction fragment (Cav1.2 1,595–1,676; Supplementary Fig. 3c–d). In both experiments, the interaction domain of Myoscape on the distal C terminus of the LTCC overlaps with the IQ motif and ranges from amino acids 1,595 to 1,676. To analyze Myoscape protein localization in cardiomyocytes, a polyclonal rabbit anti-Myoscape antibody was established (Supplementary Fig. 2). Consistent with the identified protein interactions, confocal immunofluorescence experiments of adult rat ventricular myocytes (ARVCMs) revealed colocalization of Myoscape with sarcomeric α-actinin and the LTCC (Fig. 1f,g). Colocalization of Myoscape with sarcomeric α-actinin and the LTCC was also confirmed in skeletal muscle tissue (Supplementary Fig. 4a,b), as well as colocalization of Myoscape with the cardiac ryanodine receptor 2 in ARVCMs, respectively (Supplementary Fig. 4c).

### Myoscape upregulation enhances calcium transients in ARVCM

**Figure 2:**
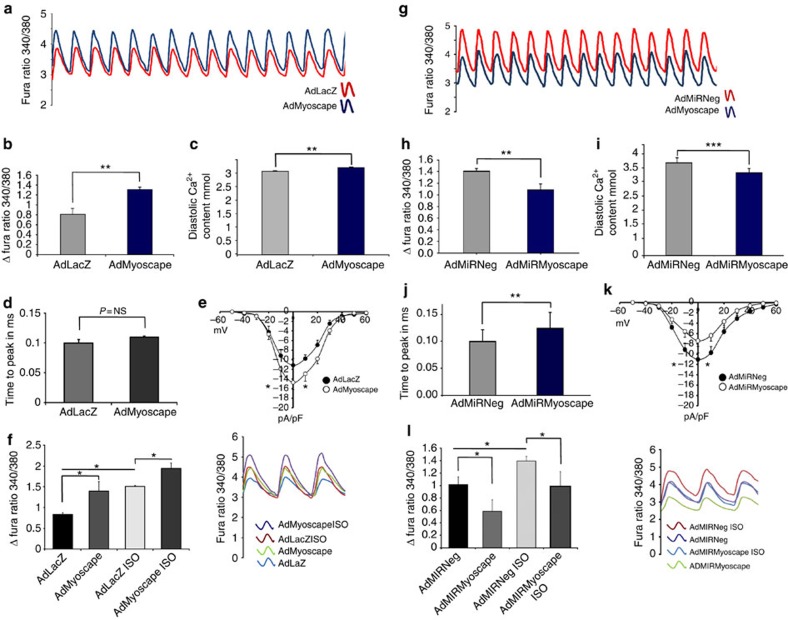
Myoscape overexpression improves global calcium transients in ARVCM. (**a**) Representative recordings of calcium transients after infection with 20 i.f.u. AdMyoscape and 20 i.f.u. AdLacZ serving as a control, and pacing ARVCM with two hertz, showing significant improvement of calcium transients on Myoscape overexpression assessed by Fura-2 ratios. (**b**) Statistical evaluation of global calcium amplitude (total count of 135 cells, *n*=4 independent experiments, **P<0.001 (ANOVA)) and diastolic calcium content (**c**); total count of 130 cells, *n*=4, **P<0.01 (ANOVA)) in ARVCMs after adenoviral Myoscape overexpression. (**d**) Time to peak measurements of calcium transients after Myoscape overexpression were unaffected compared with AdLacZ infected cells. (**e**) Measurements of whole-cell L-type calcium currents in ARVCM's after adenoviral Myoscape overexpression compared with LacZ-infected controls, (nine cells of two animals each; 20 i.f.u.; *P<0.05 (ANOVA)). (**f**) Statistical evaluation (left panel) and representative calcium transient recordings of ARVCMs (right panel) after overexpression of Myoscape in the presence or absence of simultaneous isoproterenol stimulation (100 μM). (**g**) Myoscape ablation impairs global calcium transients in ARVCM as shown by a representative recording of calcium transients assessed by Fura-2 ratios after infection of ARVCM with 100 i.f.u. AdMiRMyoscape and 100 ifu AdMiRNeg as a control (**h**) Statistical evaluation of global calcium amplitude (total count of 120 cells, *n*=4, ***P<0.001 (ANOVA)) and diastolic calcium content (**i**) in ARVCM after adenoviral Myoscape knockdown. (**j**) Statistical evaluation of the time to peak measurements in ARVCM (total count of 60 cells, *n*=3, 0.12 versus 0.09 ms, **P<0.01 (ANOVA)). (**k**) Measurements of whole-cell L-type calcium currents after adenoviral Myoscape knockdown compared with AdMiRNeg-infected controls (*n*=9cells of 2 animals each), revealing significant impairment of L-type channel function *P<0.05). (**l**) Statistical evaluation (left panel) and representative calcium transient recordings of ARVCMs (right panel) after knockdown of Myoscape in the presence or absence of isoproterenol (100 μM) (*n*=3) *P<0.05 (ANOVA). NS, not significant.

Since Myoscape interacts with the C terminus of the LTCC we hypothesized that it might modulate calcium cycling in cardiomyocytes. Infection of ARVCMs with an adenovirus encoding Myoscape (AdMyoscape) or β-galactosidase (AdLacZ; Supplementary Fig. 5a) revealed that overexpression of Myoscape enhances global calcium transients (Fig. 2a) with significantly increased Ca^2+^ amplitudes (Fig. 2b) and diastolic Ca^2+^ contents (Fig. 2c). Next, we tested whether the L-type calcium current can be directly affected by the modulation of Myoscape protein content using whole-cell patch clamp techniques. The overexpression of Myoscape in ARVCMs led to a profound increase of LTCC-dependent calcium influx (Fig. 2d), while time to peak was not significantly affected by Myoscape overexpression (Fig. 2e). When ARVCMs were co-stimulated with the β-agonist isoproterenol, a known stimulator of calcium transients, Myoscape was still able to further increase calcium fluxes, indicating that Myoscape's function is independent of beta-adrenergic stimulation and its associated downstream signalling cascades (Fig. 2f).

### Myoscape downregulation inhibits calcium transients in ARVCM

We also generated two adenoviruses encoding for a synthetic microRNA (miRNA) directed against rat Myoscape sequence (AdMiRMyoscape). An adenovirus expressing a scrambled miRNA sequence served as the control (AdMiRNeg; Supplementary Fig 5b). Infection efficiency reached >90% (Supplementary Fig. 5c). Adult cardiomyocytes and neonatal cardiomyocytes revealed a robust downregulation of Myoscape protein expression starting 24 h of infection (Supplementary Fig. 5b and 5c). After infection with AdMiRMyoscape, ARVCMs showed a significant downregulation of global calcium transients (Fig. 2g), with reduced Ca^2+^ amplitudes (Fig. 2h) and a lower diastolic Ca^2+^ content (Fig. 2i). Adenoviral knockdown of Myoscape also extended the time to peak of the calcium transients, consistent with impaired function or mislocalization of the pore-forming unit (Fig. 2j). Myoscape ablation also reduced LTCC calcium currents in ARVCMs compared with control virus-infected cells (Fig. 2k). Again, this regulation was independent of beta-adrenergic stimulation, as β-stimulation with isoproterenol could completely recover the effects of Myoscape depletion (Fig. 2l).

### Downregulation of Myoscape leads to impaired contractility

**Figure 3:**
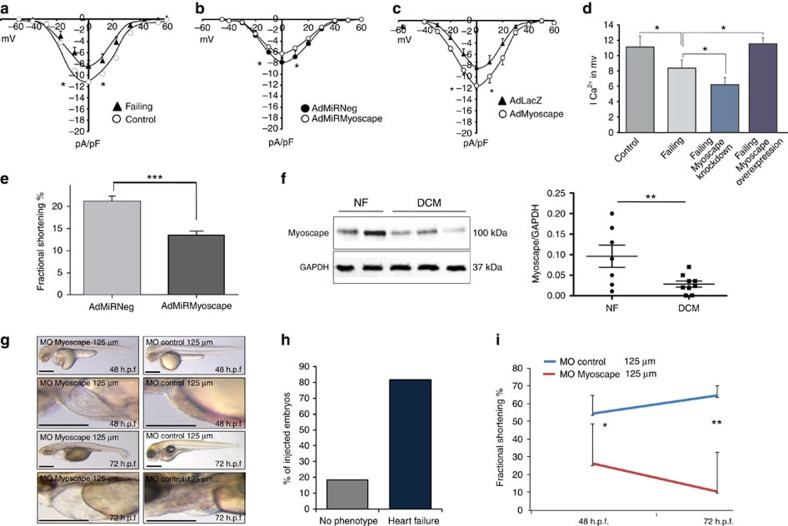
Myoscape ablation and heart failure. (**a**) Measurements of whole-cell L-type calcium currents in failing rat ARVCMs isolated from rat hearts after cryoinfarction, compared with non-failing (sham operated) controls at baseline or (**b**) after adenoviral Myoscape knockdown or (**c**) Myoscape overexpression, respectively (*n*=7–9, *P<0.05 (ANOVA)). (**d**) Summary of the L-type calcium currents under different experimental conditions, showing either restoration of calcium currents or a further reduction depending on Myoscape protein content. (*n*=7, *P<0.05 (ANOVA)) (**e**). To elucidate the functional consequences of myoscape downregulation on cardiomyocyte contractile function, again adult ventricular rat cardiomyocytes were utilized. On the first day of culture, ARVCMs where infected with AdMiRMyocape or control virus (AdMiRNeg). Fractional shortening was assessed by video edge detection evaluated by an observer blinded to experimental conditions. ARVCMs infected with AdMiRMyoscape showed a significant reduction in fractional shortening compared with ARVCM's infected with control virus (21±1% versus 14±1%, in 60 assessed cells, and *n*=3 independent cell preparations, ***P<0.001 (ANOVA)). (**f**) Downregulation of Myoscape protein content was shown via western blot analyses in biopsies from patients suffering from end-stage heart failure due to dilated cardiomyopathy (DCM) and compared with control heart samples from individuals who died from a non-cardiovascular-cause (*n*=7–8). GAPDH served as a loading control (left panel). A statistical evaluation was done of all samples showing a significant decrease of Myoscape/GAPDH ratios. **P<0.01 (ANOVA). (**g**) Morpholino knockdown of zebrafish Myoscape results in severe contractile dysfunction. Myoscape knockdown morphants (lateral view) develop pericardial oedema and blood congestion due to low cardiac performance. By contrast, cardiac function of MO-control injected zebrafish embryos was unaffected after 48 or 72 h.p.f. (**h**) Quantitative evaluation showing 81.7% of Myoscape knockdown morphant zebrafish developing cardiac failure after injection. The presence of heart failure was assessed by evaluation of reduced ventricular function and development of pericardial oedema and precardial signs of blood congestion. Functional assessment of cardiac contractility was carried out by a Zeiss MCU II microscope with the help of the zebraFS software. (**i**) Fractional shortening (FS) of the ventricular chamber of wild-type and Myoscape-deficient embryos measured at different developmental stages (48 and 72 h.p.f.). *P<0.05; **P<0.01 (ANOVA). Scale bars, 25 μm.

We next asked whether Myoscape is able to modulate calcium fluxes in cardiomyocytes under conditions of experimental heart failure. We thus examined ARVCMs from rat hearts in which heart failure was induced using an experimental post-cryoinfarction heart failure model^26^,^27^. These cells show a reduction in calcium currents compared with cells isolated from sham-operated hearts (Fig. 3a). The knockdown of Myoscape further impaired LTCC-dependent calcium currents (Fig. 3b), while the reduction in calcium currents observed in failing cardiomyocytes could be reversed on Myoscape overexpression (Fig. 3c, summarized in Fig. 3d). Consistent with impaired calcium cycling, ARVCMs infected with AdMiRMyoscape showed a significant reduction in fractional shortening compared with ARVCMs infected with control virus (21±1% versus 14±1%, in 60 assessed cells, and *n*=3 cell preparations, ***P<0.001 (analysis of variance (ANOVA); Fig. 3e).

To examine whether Myoscape expression is altered in disease states we first analysed myocardial tissue from chronically infarcted pig hearts^28^. Interestingly, Myoscape expression was not significantly different in comparison with sham controls (Supplementary Fig. 5d), suggesting that the observed reduction in LTCC currents on myocardial infarction cannot simply be explained by myoscape downregulation. Next we assessed whether Myoscape expression is differentially regulated in end-stage human heart failure. Therefore, we analysed myocardial samples from eight patients undergoing cardiac transplantation due to advanced dilated cardiomyopathy. Seven samples from non-failing hearts served as controls. Of note, a significant −80% downregulation of Myoscape protein could be detected in human cardiomyopathy (**P<0.01 using ANOVA test, Fig. 3f).

### Functional consequences of a Myoscape knockdown *in vivo*

To assess the function of myoscape *in vivo*, we next injected zebrafish embryos with morpholino-modified antisense oligonucleotides directed against the splice site exon 2/intron 2 (E2I2) of the zebrafish Myoscape orthologue (Supplementary Fig. 6a), leading to skipping of exon 2 as revealed by splice site analysis, predicted toy result in premature termination of protein translation. When injected with 4 ng of MO-Myoscape, 81.7% of embryos (total of *n*=63 injected embryos) revealed contractile dysfunction and heart failure (Fig. 3g,h and Supplementary Movie 1) compared with controls (Fig. 3g, Supplementary Movie 2). In Myoscape knockdown morphants, ventricular fractional shortening was drastically decreased to 24% (48 h) and 10% (72 h; Fig. 3i), resulting in decreased blood flow, precardial congestion and pericardial effusion.

To evaluate whether Myoscape deficiency interferes with key steps of zebrafish heart development, we examined the expression of cardiac chamber-specific proteins and messenger RNAs (mRNAs) by immunostainings and antisense RNA *in situ* hybridization, respectively, as well as the cardiac structure of Myoscape morphants by histology^29^,^30^. While minimal residual Myoscape expression cannot be excluded, knockdown of Myoscape did not interfere with crucial steps of cardiogenesis, such as heart tube looping, chamber demarcation and the differentiation of ventricular and atrial cardiomyocytes (Supplementary Fig. 6b). Likewise, the expression of cardiac chamber-specific myosin heavy chains (MF20, S46) was found in the correct heart chamber restricted pattern (Supplementary Fig. 6c). We also analysed the ultrastructure of zebrafish cardiac muscle cells by light microscopy (Supplementary Fig. 6d) as well as by transmission electron microscopy. Again, at 72 h.p.f., Myoscape-deficient morphants showed no ultrastructural defects in t-tubule, sarcoplasmic reticulum (SR) and Z disc architecture, as well as in cardiac and skeletal muscle sarcomeres (Supplementary Fig. 6e and f).

### Myoscape KO mice display cardiac contractile dysfunction

**Figure 4:**
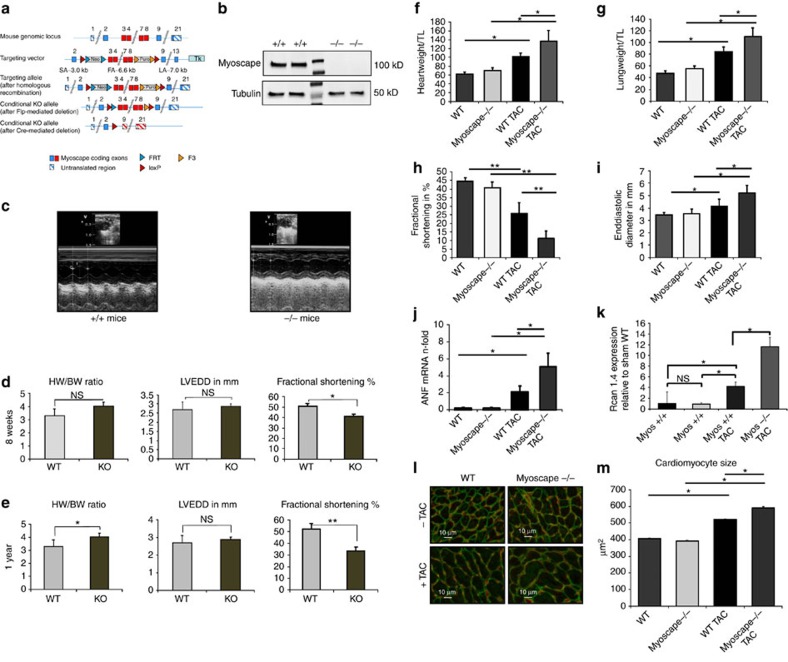
Strategy and cardiovascular phenotyping of Myoscape KO mice. (**a**) Schematic representation of the knockout strategy resulting in a constitutive knockout after loxP-Cre-mediated deletion of exons 3–8. (**b**) Western blot analysis confirming that Myoscape −/− mice do not express detectable Myoscape protein at the expected size of 105kD. The mid-lane represents marker protein. (**c**) Representative echocardiographic recordings by a blinded observer from Myoscape +/+ mice at the age of 6–8 weeks and from Myoscape −/− mice at the age of 6–8 weeks. (**d**) Statistical evaluation of morphometric (HW/BW) and echocardiographic (LVEDD and FS in %) parameters from Myoscape −/− or +/+ mice at the age of approx. 8 weeks and (**e**) after one year revealing progressive heart failure in the absence of severe hypertrophy. *P<0.05; **P<0.01. (ANOVA) (**f**) After 4 weeks of pressure overload due to transverse aortic constriction (TAC), Myoscape knockouts showed a significant increase of heart weight to tibia length ratios compared with wild-type mice (136.6±24 versus 101.1±9; *P<0.05 (ANOVA)). (**g**) Myoscape-null mice show higher lung weight to tibia length ratios (110±15 versus 84±8.5; *P<0.05 (ANOVA)) than control littermates, indicating the presence of congestive heart failure. (**h**) In echocardiographic measurements of ventricular fractional shortening, Myoscape knockouts show significantly exacerbated contractile impairment (11.2±6.4 versus 25.6±4.4; *P<0.05 (ANOVA), further underlined by significant ventricular enlargement. (**i**) Absence of Myoscape leads to a nduction of the hypertrophy-associated fetal gene programme as assessed by qPCR analysis of significantly increasd ANF levels and a strong induction of Rcan 1.4 levels as compared with wild-type animals (**j**,**k**), and also leads to a more pronounced increase of cardiomyocyte cell size after transverse aortic constriction (**l**,**m**). (592 μm^2^± 6.3 s.e.m. versus 519 μm^2^ ±5.1 SEM; *P<0.05 (ANOVA)). NS, not significant.

Next, we engineered a Myoscape KO mice line via loxP-mediated disruption of exons 3–8 (Fig. 4a and Supplementary Fig. 11). Absence of Myoscape protein expression was confirmed by western blot analyses (Fig. 4b). Histochemical analyses and electron microscopy revealed no apparent signs of structural cardiomyocyte defects (Supplementary Fig. 7a–c). Myoscape KO mice were viable and fertile and revealed normal blood pressure and heart rates (Supplementary Fig. 7d–e). In contrast, cardiac function, as assessed *in vivo* by echocardiography (Fig. 4c), was found to be significantly impaired in Myoscape KO mice with a reduction of fractional shortening after 8 weeks, which further progresses after one year (FS 51.7±4.2% versus 33.3±3.7%, *n*=10). Myoscape-deficient hearts showed no significant ventricular enlargement, and only a non-significant trend towards an increased heart weight to body weight ratio under basal conditions (Fig. 4d,e). Interestingly, echocardiogram recordings of Myoscape-null mice revealed a significant shortening of the QT and the corrected QTc interval in females compared with wild-type mice (Supplementary Fig. 8), similar to human patients with LTCC loss of function mutations^31^. A similar trend was also seen in male littermates, but did not reach statistical significance.

### Pressure overload worsens heart failure in Myoscape knockouts

In contrast to the basal phenotype, after 4 weeks of pressure overload due to transverse aortic constriction (TAC), Myoscape KO animals showed an excessive increase of heart weight to tibia length ratios as compared with wild-type mice subjected to aortic constriction (136.6±24 versus 101.1±9; *P*<0.05 (ANOVA); Fig. 4f). Moreover, Myoscape-null mice revealed exacerbated pulmonary congestion after TAC, as shown by higher lung weight to tibia length ratios (110±15 versus 84±8.5; *P*<0.05 (ANOVA); Fig. 4g). On pressure overload, we also observed severely impaired fractional shortening (11.2±6.4 versus 25.6±4.4; *P*<0.05 (ANOVA); Fig. 4h) as well as an increase of end-diastolic diameters (Fig. 4i) and a trend towards increased end-diastolic volumes, respectively (Supplementary Fig. 9a). The absence of Myoscape led to ‘super-induction' of the fetal gene program member ANF, which was not seen under unchallenged conditions (Fig. 4j and Supplementary Fig. 9b and c). Likewise, we observed an additional increase of cardiomyocyte cell size (592 μm^2^±6.3 s.e.m. versus 519 μm^2^±5.1 s.e.m.; *P*<0.05 (ANOVA); Fig. 4l,m) in response to TAC. Cardiomyocyte hypertrophy was accompanied by a strong induction of RCAN1.4 mRNA (Fig. 4k) and protein (Supplementary Fig. 9d) expression, indicating excessive activation of calcineurin/NFAT signalling in Myoscape KO hearts in response to increased biomechanical stress^32^. Consistently, Myoscape KO mice showed enhanced fibrosis after aortic constriction, as shown by sirius red stainings of heart sections (Supplementary Fig. 9e) mRNA levels of collagen I and III showed a strong trend towards increased expression (Supplementary Fig. 9f–g).

### Myoscape regulates LTCC plasma membrane targeting

**Figure 5:**
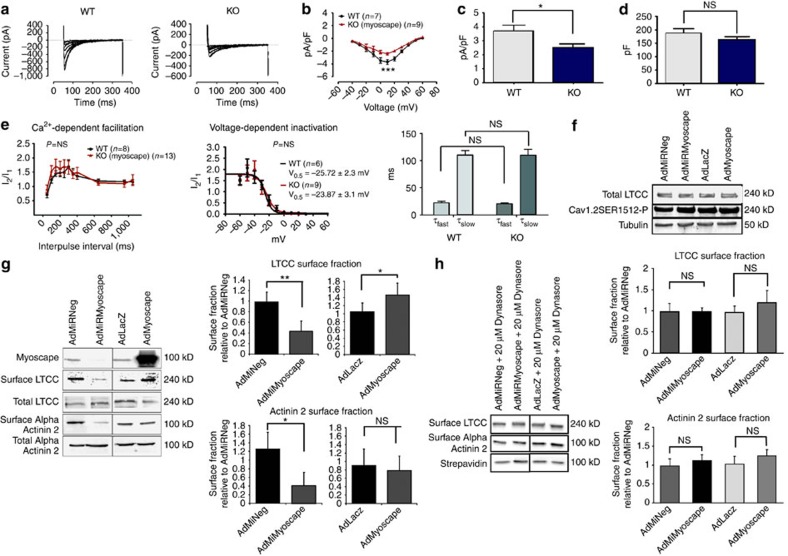
Myoscape regulates LTCC surface retention. (**a**) Electrophysiological measurements of ventricular cells from Myoscape KO and WT mice showing impaired LTCC currents. Cells were stimulated by the voltage protocol. A prepulse of −80 to −40 mV was used to inactivate fast Na+ currents. Current traces and voltage protocols are superimposed from −40 to +60 mV. Current–voltage relation of ICa Peak current density was plotted against the voltage pulse. (**b**) IV curve analysis showed a highly significant reduction of L-type currents in Myoscape KO mice (*P*<0.0001; (ANOVA)). (**c**) Myoscape KO mice showed reduced current densities (3.72±0.4 versus 2.48±0.2; *P<0.05 (ANOVA)) and (**d**) a strong trend toward reduced cell capacitance (187.1±17 versus 161.2±12, *P*=NS (ANOVA)). (**e**) Facilitation of ICa (CDF) was measured by a twin-pulse protocol. Current traces obtained at time intervals (Δt) of 50–350 ms in 50-ms increments are superimposed fractions of current (I2/I1) and are plotted against the interval duration (Δt). Voltage-dependent inactivation (VDI) was measured using the IV protocol. Right panel shows time constants T for fast and slow inactivation of the LTCC. Both CDF/VDI and T constants were unaltered comparing WT and KO cells. (**f**) Western blot showing Cav1.2 phosphorylation of serine residue 1512 with adenoviral knockdown or overexpression of Myoscape in NRVCMs using a phosphor-specific antibody from Covalab (pab0901; P Cav1.2 p1512). No significant differential phosphorylation could be detected in the absence or presence of Myoscape. (**g**) LTCC membrane expression was assessed by a surface biotinylation assay (Pierce) in NRVCM's and was compared with total cellular LTCC content and normalized to total streptavidin. Myoscape knockdown by infection of NRVCM's with AdMiRMyoscape (100 ifu) significantly decreased LTCC and α-Actinin surface expression, whereas overexpression of Myoscape protein (with 50 ifu AdMiRMyoscape) increased LTCC surface density as compared with control virus infection (t-test; *P*<0.05). In contrast, whole-cell LTCC and Actinin content were unaffected. *P<0.05; **P<0.01. ANOVA (**h**) LTCC protein and alpha-actinin 2 membrane expression was again assessed by the surface biotinylation assay in NRVCM's together with the endocytosis inhibitor Dynasore (20 μM). In the presence of Dynasore, Myoscape knockdown no longer decreased LTCC and alpha-actinin surface expression. NS, not significant.

To unravel the mechanism of altered calcium metabolism, Myoscape-deficient cardiomyocytes derived form Myocape KO mice were next analysed by whole-cell patch clamp experiments (Fig. 5a). Consistent with the earlier findings in AdMiRMyoscape transduced ARVCMs, Myoscape-null cardiomyocytes showed reduced L-type Ca^2+^ currents (*P*<0.0001; *n*=7–9 (ANOVA)) and current densities (pA/pF 3.72±0.4 versus 2.48±0.2, *P*<0.05 (ANOVA); Fig. 5b–d). Moreover, these cells showed a strong trend towards reduced overall cell capacity (187.1±17 versus 161.2±12, *P*=0.07 (ANOVA)). Yet, surprisingly, both voltage-dependent inactivation (VDI) and CDF were unaffected, suggesting that activity of the individual LTCC was not differentially regulated (Fig. 5e). Consistently, CaMKII-dependent phosphorylation of SER1512 (which is thought to indicate CaMKII-CDF) was not altered in Myoscape-null mice (Fig. 5f). Likewise, time constants T for slow and fast LTCC inactivation were not affected in Myoscape KO hearts compared with wild-type controls, suggesting that CDI was also not impaired (Fig. 5e, right panel). Of note, we also did not observe any differences in overall α-actinin 2 or LTCC protein content in heart samples from KO mice compared with wild type (*n*=10, Supplementary Fig. 10a and b). Finally, SERCA 2a mRNA and protein levels were also not differentially regulated in the presence or absence of Myoscape (Supplementary Fig. 10c and d).

In light of these findings we hypothesized that Myoscape might instead influence calcium currents via altered LTCC surface expression and microdomain function. To test this, we again infected NRVCMs with ADMiRMyoscape and AdMyoscape and assessed LTCC and α-actinin 2 membrane association by surface biotinylation assays. In these experiments, Myoscape knockdown significantly decreased LTCC and also α-actinin 2 surface expression (Fig. 5g, left and right panels), while overexpression revealed an increased membrane-bound LTCC content. Remarkably, the reduction of membrane LTCC content due to Myoscape depletion was completely abolished by treatment with the endocytosis inhibitor Dynasore, indicating that the observed LTCC surface reduction critically depends on endocytosis (Fig. 5h).

## Discussion

Here we present the detailed molecular and functional characterization of the gene *Myoscape/FAM40B/STRIP2*, encoding a protein abundandtly expressed in striated muscle tissue. Previously, Myoscape/FAM40B has been described as a member of a novel large multiprotein complex of striatin-interacting phosphatases and kinases associated with PP2A and cerebral cavernous malformation protein 3 (ref. 25). Yet, so far, no specific function could be attributed to Myoscape/FAM40B. We now provide evidence that Myoscape plays a key role in regulating cardiomyocyte calcium currents and cardiac contractility under physiological and pathophysiological conditions via stabilization of LTCC surface expression.

Despite decades of research, heart failure remains a major cause of death and mortality^32^,^33^,^34^,^35^,^36^,^37^,^38^. Therefore, improved understanding of the molecular mechanisms underlying heart failure is still urgently needed to develop novel targeted treatment strategies. Thus, to search for new molecular targets affecting cardiac function, we took an *in silico* approach to identify novel genes mainly expressed in cardiac tissues^24^. mRNA and protein expression analyses confirmed an enrichment of Myoscape in human and murine cardiac tissue. A large fraction of Myoscape was detected in the sarcolemma at the t-tubule/z-disc interface, where it interacts and colocalizes with α-actinin 2 and the LTCC. Interaction studies revealed that the interaction of Myoscape with the distal C terminus of the LTCC ranges from amino acids 1,595 to 1,676 and thus directly overlaps with a recently reported interaction domain between α-actinin 2 and the LTCC (amino acids 1,584–1,670) in neurons^39^. Previous studies revealed that the ‘cardiac calcium release unit' represents a multiprotein complex involving various regulatory and scaffolding components that are crucial in modulating LTCC pore activity, channel microdomain localization and associated signalling^40^. In this context, the overexpression of Myoscape resulted in enhanced global calcium transients with higher calcium amplitudes and LTCC currents. These results were additive to the effects of isoproterenol, implying that Myoscape acts independently of the adrenergic control of LTCC activity via PKA-dependent phosphorylation^9^,^13^,^26^. Conversely, Myoscape ablation reduced LTCC currents irrespective of beta-adrenergic costimulation, which is associated with reduced global calcium transients and impaired contractile performance of cardiomyocytes.

The opening of the LTCC, representing the key calcium entry regulator, is tightly controlled by various mechanisms, usually involving the channel C-terminal tail^11^,^19^,^41^,^42^. Regulatory cascades can be divided into short-term responses, like conformational changes due to protein–protein interactions or phosphorylation, and long-term regulatory pathways, such as transcriptional regulation and membrane targeting^2^. Regarding short-term regulation, the LTCC pore unit shows few VDI components, but it is rather regulated by calcium itself through CDI) and CDF, respectively^16^,^41^,^42^. Many of these processes are thought to represent ‘safety' mechanisms preventing calcium overload with deleterious consequences including cell death^3^. Cardiomyocytes from Myoscape KO mice still showed proper VDI and no relevant alterations of CDF, suggesting that Ca-calmodulin-dependent LTCC regulation is unaltered. This was further underlined by the finding that Myoscape did not differentially affect CamKII-dependent phosphorylation of the distal-C-terminus (DCT) at S1512, which mediates calcium current facilitation in the murine heart^41^,^43^.

In the absence of a direct regulatory effect on LTCC function, we next explored differential membrane localization of the LTCC in the absence or the presence of Myoscape as a potential mechanism. Of note, LTCCs undergo continuous replacement with newly synthesized channels with a short half-life of 3–4 h (refs 21, 22). This replacement offers additional regulatory options via internalization, degradation and storage of channels in vesicles, as well as in delocalization and lateral diffusion of LTCCs^3^. So far, LTCC surface regulation has been studied mostly in neurons and in heterologous cell systems, and it involves a variety of interacting proteins or auxiliary subunits. For example, the LTCC interacting protein eLF3e was reported to promote a calcium-sensitive endocytotic signal in neurons, leading to reduced LTCC surface expression^44^. Interestingly, the neuronal plasma membrane expression of LTCCs has recently been reported to be controlled by the physical interaction of α-actinin with the IQ motif on the distal C terminus of the channel^39^. In a hypothetical model, calcium-free calmodulin binds with α-actinin to the IQ motif, whereby α-actinin tethers the LTCC to cortical F-actin. Calcium influx results in the saturation of ApoCalmodulin with calcium and subsequent short-term CDI. Persistent calcium influx then displaces α-actinin from the channel, leading to LTCC internalization^39^. Given the fact that in cardiomyocytes the C terminus of the calcium channel seems to be crucial for correct t-tubule targeting and LTCC surface density, and given that Myoscape and α-actinin share an almost identical interaction domain on LTCCs (see above), we tested whether Myoscape was required for LTCC surface retention in cardiomyocytes. These experiments revealed that Myoscape ablation indeed significantly reduces LTCC surface expression. Moreover, this reduction of surface LTCC expression depends on endocytosis as the endocytosis inhibitor Dynasore abolished the effects of Myoscape depletion on LTCC membrane localization. Consistent with these results, immunohistological studies also showed a reduction of t-tubule-associated LTCC expression in ARVCMs.

Taken together, our data thus support a concept where Myoscape stabilizes LTCC surface expression by promoting an interaction with the LTCC and alpha-actinin at the t-tubule. Consistently, Myoscape overexpression further enhances LTCC surface stability and calcium currents in both normal cells and even failing cardiomyocytes, leading to increased or restored calcium cycling properties. The loss of Myoscape results in disruption of this membrane-associated complex, resulting in LTCC internalization and reduced cardiac calcium channel currents both *in vitro* and *in vivo*. Of note, Myoscape-deficient female mice also reveal a significant shortening of the QT interval. In humans, this phenotype has been associated with loss of function mutations of the LTCC alpha-subunit encoding *CACNA1C* gene^42^, further supporting the concept that the findings in myoscape-deficient mice can be explained by LTCC impairment. Interestingly, one of these mutations (A39V) also led to reduced membrane localization of the LTCC^42^.

One well-known hallmark of failing heart cells is disturbances in calcium cycling^45^,^46^. For example, the expression of SR-bound SERCA2 is consistently downregulated in heart failure, while the activity of the sarcolemmal sodium–calcium exchanger (NCX) is typically upregulated^34^. Many of these alterations can be viewed as a short-term effort to remove harmful calcium from cardiomyocytes, yet at the cost of sustained deterioration of contractile function^4^. Interestingly, we also observed a downregulation of Myoscape protein content in heart muscle biopsies of end-stage dilated cardiomyopathy patients, potentially representing a new mechanism to protect the failing cardiomyocyte against calcium overload. In line with this notion, Nakayama *et al*.^46^ could show that overexpression of the LTCC in transgenic mouse hearts leads to extended cardiomyocyte necrosis, pump failure and premature death. Yet, the price to pay for short-term protection against calcium overload may be further (long-term) deterioration of contractile function. Consistent with our *in vitro* data and similar to recent data by Wagh *et al*.^47^, loss of Myoscape/FAM40B indeed severely reduced the contractile performance of zebrafish hearts *in vivo*, resulting in progressive cardiomyopathy. Likewise, Myoscape KO mice revealed a deficit of cardiac contractility in the absence of relevant hypertrophy or structural alterations. Notably, we could not detect any differential regulation of major calcium handling proteins like the LTCC itself or SERCA2A.

The contribution of LTCC calcium currents in the development and the progression of heart failure nevertheless still remains controversial. While several groups have shown that LTCC currents are reduced in both experimental^48^ and human^49^,^50^,^51^ heart failure, others reported only little differences^52^. In this context it is important to note that LTCC currents not only initiate excitation–contraction coupling in cardiac muscle, but have also been implicated in calcium-dependent signalling in cardiomyocytes. For example, caveolae-associated LTCCs have been shown to mediate pathological hypertrophy, while their specific inhibition did not affect contractile function^10^. Localization of LTCCs to this caveolae-associated macromolecular complex are also required for proper beta-adrenergic signalling in cardiomyocytes^9^. Mice with severely decreased L-type channel activity developed hypertrophy and heart failure^53^, presumably via the activation of compensatory transcriptional pathways. Heterozygous (*Cacna1C*) LTCC KO mice only developed significant hypertrophy when subjected to additional biomechanical stress (TAC). The concluding hypothesis of the groups of Bers and Molkentin^53^ was that, in the absence of a sufficient LTCC current, SR Ca^2+^ release becomes sensitized to maintain cardiac contractility, in turn leading to hypertrophic remodelling through calcineurin/NFAT activation. In line with this theory, *Ncx1* KO mice, which show up to 60% reduction in LTCC currents, also show exaggerated hypertrophy after TAC^54^. These results completely mirror our data in Myoscape-deficient mice, which also did not show relevant hypertrophy at baseline yet revealed significantly enhanced, and calcineurin/NFAT-dependent, pathological hypertrophy with increased heart weights and cardiomyocyte cell size when subjected to additional biomechanical stress. This maladaptive cardiac hypertrophy was associated with severely impaired left ventricular performance. In contrast, a recently published Polycistin 1 (*Pc-1*)-KO mouse model, shows less LTCC surface retention and diminished Ca currents, and revealed attenuation of the hypertrophic response after TAC^55^. One potential explanation for these apparent discrepancies might be that LTCC function (prohypertrophic signalling versus excitation-contraction (EC) coupling) depends on its specific subcellular localization in microdomains which could be differentially affected by LTCC-associated proteins such as PC-1, BIN1 (refs 51, 55) and Myoscape. In summary, we identified the cardiac-enriched protein Myoscape as a new regulator of LTCC membrane expression and cardiomyocyte contractile force generation. This novel molecular pathway adds another layer of regulation to calcium metabolism in cardiomyocytes with implications for the molecular understanding of calcium signalling as well as EC coupling in heart failure and cardiomyocyte hypertrophy.

## Methods

### Cloning of human and rodent Myoscape/FAM40B

The complete ORFs of murine Myoscape were amplified from cardiac cDNA of the corresponding species employing the Gateway technology (Invitrogen). The following gateway compatible and gene-specific primers were used, MyoscapeF and MyoscapeRs respectively:

MyoscapeF with start codon

(5′-GGGGACAAGTTTGTACAAAAAAGCTGGCACCatggaggaccccgc-3′)

MyoscapeR with stop codon

(5′-GGGGACCACTTTGTACAAGAAAGCTGGGTCGCCtcagtgattctggagcagctc-3′)

MyoscapeR without stop codon

(5′-GGGGACCACTTTGTACAAGAAAGCTGGGTCGCCgtgattctggagcagctc-3′)

For integration of the specific recombination site into the PCR-products, a second universal PCR was performed using attBFor (5′-GGGGACAAGTTTGTACAAAAAAGCTGGCACC-3′) and attBRev (5′-GGGGACCACTTTGTACAAGAAAGCTGGGTCGCC-3′) primers and 1 μl of the initial PCR reaction as template. The PCR product containing the recombination-specific attachment sites (att-sites) was further recombined into the pDON201 entry vector.

For recombinant protein expression, rat Myoscape cDNA was subsequently shuttled into Gateway compatible expression plasmids to obtain expression constructs encoding for amino-terminal fusion tags (HA; MYC).

### Cloning of synthetic Myoscape knockdown miRNAs

Knockdown Oligonucleotides miRMyoscape_Top (5′-TGCTGTAGAGGATTTCACTCGGTGTTGTTTTGGCCACTGACTGACAACACCGAGAAATCCTCTA-3′) and miRMyoscape_Bottom (5′-CTTGTAGAGGATTTCTCGGTGTTGTCAGTCAGTGGCCAAAACAACACCGAGTGAAATCCTCTAC-3′) were designed using Invitrogen's BLOCK-iT RNAi Designer and subsequently cloned into the pcDNA6.2-GW/EmGFP-miR vector according to manufacturer's instructions. This construct covered a 20 nucleic acid Myoscape mRNA sequence, directed against a homologous sequence part in mouse and rat, and it was used as template for the recombination into the pDON201 entry vector which itself served as template for the generation expression constructs. As negative control we used the pcDNA6.2-GW/EmGFP-miR plasmid, which can form a hairpin structure and is consecutively processed into a mature miRNA, yet is predicted not to target any known mammalian gene (sequence of the insert: 5′-GAAATGTACTGCGCGTGGAGACGTTTTGGCCACTGACTGACGTCTCCACGCAGTACATTT-3′).

### Generation of recombinant adenoviruses

Adenoviruses were generated using the appropriate Entry vectors in combination with the ViraPower Adenoviral Expression System (Invitrogen) according to the manufacturer's instructions. Ad-β-galactosidase-V5 or an adenovirus encoding for a miRNA backbone but not carrying any predicted binding sites known to target any mammalian gene (AdMiRNeg) served as control for experiments (Invitrogen). 2 h after plating, adult rat cardiomyocytes infected with an indicated adenoviral construct for at least 24 h.

### Northern blot analysis

Multiple tissue northern blots (Clontech and Origene) containing mouse or human poly(A) RNA were hybridized overnight at 65 °C with ^32^P-dCTP-labelled (Rediprime II Random Prime labelling System, Amersham Biosciences) cDNA probes corresponding to the ORF of mouse and human Myoscape/FAM40B, respectively. The probe covered Myoscape's sequence from approximately 1,076–1,300. Serial washes were conducted with 2 × SSC/0.1% SDS and 0.2 × SSC/0.1% SDS at 65 °C. Autoradiography was performed at −80 °C for 24–168 h with an intensifying screen. Primers used to generate the Northern Blot probe:

Myoscape Forward (fwd): (5′-TCCGCCTTCTTACACTCTTGA-3′)

Myoscape Reverse (rev): (5′-TGCGATGGAGATGTACTTGTG-3′)

### Y2H library screening

Automated yeast two-hybrid screens were performed^56^. Myoscape was divided into two expression construct expression one C-terminal fragment and one N-terminal fragment that were then fused to a GAL4 DNA-binding domain (in pDEST32, Invitrogen). The constructs were used as baits in a yeast two-hybrid screen of ∼1 × 10^6^ clones. Human cDNA libraries from human heart and skeletal muscle (Clontech) as well as a library of individually cloned full-length ORFs from cDNAs of 5,000 different genes were screened to a minimal coverage of five million clones per library. To mate yeast strains harbouring the bait protein and the prey library, exponentially growing cultures of an OD_600nm_ of 1 were combined, pelleted by centrifugation for 2 min at 2,900 r.p.m., and resuspended in an equal volume of YPDA containing 20% PEG 6000 in a 50 ml centrifugation tube. Mating mixes were incubated at 30 °C with gentle agitation (100 r.p.m.) for exactly 3 h, before washing and resuspending the cells in selective medium. For the generation of a high-confidence data set, interaction pairs were selected which were isolated at least twice, or where the bait interacted with two highly related preys, and which did not involve promiscuous preys.

Primers for Y2H Constructs were:

Myoscape CT1R: 5′-GCTGGGTCGCCTCAAGCTTAGAGCTTTGGGAAG-3′

Myoscape CTF2: 5′-GCTGGCACCATGAACGATGACTGGGCTTAC-3′

Myoscape 1F: 5′-GCTGGCACCATGGACGACCCCGCG-3′

Myoscape 1R: 5′-GCTGGGTCGCCTTAGTGATTCTGGAGCAGCTCC-3′

### Co-immunoprecipitation experiments

Interacting proteins were transiently coexpressed in HEK293-T cells as N-terminal fusions. 48 h after transfection, with jetPEI (PolyPlus Transfection) according to manufacturer instructions, the medium was removed and cells were lysed in 1,000 μl of ice-cold ELB lysis buffer (50mM HEPES pH 7.0, 250 mM NaCl, 1% NP40, 5 mM EDTA, Protease Inhibitor Cocktail (Roche # 1836170) and Phosphatase Inhibitor Cocktail 1+2 (Sigma # P2850; P5726). Lysates were incubated on ice for 30 min. Cellular debris was removed by centrifugation, 5 min at 13,000 r.p.m. 4 °C. 3,000 μl of the cleared protein extract was incubated together with 50 μl Anti-HA-Agarose (Sigma # A2095) for 2 h at 4 °C with moderate agitation. After four washes with 1 ml lysis buffer, the protein complexes were eluted in 1 bead volume 1 × SDS sample buffer. For western blot experiments, the beads were separated by 2 min centrifugation at 13,000 r.p.m./room temperature (RT) and the protein containing supernatant was analysed.

### Immunoprecipitation of endogenous proteins

Immunoprecipitation of endogenous proteins was performed with total protein extracted from mouse left ventricles using Dynabeads (Thermofisher Scientific) and an anti-Cav1.2 antibody (rabbit polyclonal; Novus Biologicals 1:500), whereas an anti-V5 antibody (rabbit polyclonal; Life Technologies 1:1,000) served as a control. Eluted protein was visualized by immunoblotting using anti-Myoscape (1:100) and anti-Cav1.2 antibodies (1:250).

### GST pull-down assay

GST pull-down assays were carried out using Pierce GST Protein Interaction Pull-Down Kit (Thermofisher Scientific) using the manufacturer's instructions. Briefly, Myoscape was cloned into pDest15 to produce N-terminal GST-tagged Myoscape, whereas the desired domains of LTCC/Cav1.2 were cloned in pDest17 to produce respective N-terminal 6 × His-tagged proteins. All the proteins (except for full-length Cav1.2, which was expressed in HEK cells) were overexpressed in a BL21 *Escherichia coli* strain with the induction of 0.1% L-arabinose. Cell pellets were treated with the lysis buffer provided with the kit and eluted protein was determined by immunoblotting using anti-GST (1:500)/anti-His (1:500)/anti-Cav1.2 antibodies (1:500), respectively.

### Co-IP in HEK293A cells

HEK293A cells were co-transfected with Myc-tagged Myoscape (or haemaglutinin (HA)-tagged Myoscape) and untagged Cav1.2 (Co-transfection of Cav1.2 with respective HA-/Myc-tagged empty vectors served as controls). Protein extracted from these cells was immunoprecipitated with anti-HA-/Myc-tagged magnetic beads (Biomol Research Laboratories) and detected by immunoblotting using anti-HA/anti-Myc/anti-Cav1.2 antibodies.

### Quantitative real-time PCR

cDNA was generated from total RNA using the Superscript III first strand kit (Invitrogen # 18080-051)^57–58^. The following primers were used for quantitative PCR.

CAV1.2 fwd5′-CTCCTGCAGAGAAGCCATTC-3′

CAV1.2 rev 5′-CTGAAATCAAGACCGCTTCC-3′

Myoscape fwd 5′-TCTAGCTCAAGGCACTTTCG-3′

Myoscape rev 5′-CATGACACTCAGCAGTACCC-3′

Rpl32_e1 5′-GGTGGCTGCCATCTGTTTTACG-3′

Rpl32_e3 5′-CCGCACCCTGTTGTCAATGC-3′

BNP fwd 5′-GCAGCATGGATCTCCAGAAGG-3′

BNP rev 5′-CTGCAGCCAGGAGGTCTTCC-3′

Rcan1_mFW: 5′-TAGCTCCCTGATTGCTTGTG-3

Rcan1_mREV: 5′-GGATTCAAATTTGGCCCTGG-3′

Rcan1_mPRB: 5′-ACGATGATGTCTTCAGCGAAAGTGAGAC-3′

### Western blot analyses

To determine tissue specificity of Myoscape expression, different protein extracts of rat tissues were prepared in RIPA-buffer (10 mM Tris-HCl pH 7.5, 15 mM Na2EDTA;1% NP-40;0.5% sodium deoxycholate; 0.1% SDS; Protease Inhibitor Cocktail (Roche # 1836170). After homogenization 150 μg of the cleared supernatant were separated on a 12.5% PAA-Gel and transferred on a PVDF-Membrane. The membrane was probed as indicated with a polyclonal anti-Myoscape antibody generated in rabbit (Eurogentech; Peptide EPO70153 as immunogen) or an anti-human FAM40B antibody (HPA019657 Sigma) at a dilution of 1:200 according to the manufacturer's protocol followed by ECL detection (GE Healthcare RPN2106). For mouse LTCC western blots an Anti-Cav1.2 calcium channel, NeuroMab clone L57/46 antibody was used (1:200). For rat heart material, we used the Anti-CACNA 1 C Antibody from Novus Biologicals (NBP1-42820 (1:250)). The monoclonal anti-alpha-actinin 2 antibody was purchased from SIGMA and was used in a dilution of 1:1,000.

### Immunofluorescence microscopy

The subcellular localization of Myoscape was determined in ARVCMs and in cryosections of mouse and rat cardiac tissue using indirect immunofluorescence. Adult mouse and rat cardiomyocytes were freshly prepared as described in the sections below. ARVCM were fixed in 3.4% PFA for 10 min at RT, 16h after isolation. Blocking and permeabilization was done in 5% BSA and 0.1% Triton X-100 (Sigma # T8787) for 1 h at RT. Cryosections were blocked in 5% BSA but to stain membrane proteins, were not permeabilized with Triton.

For detection, the cover slips or slides, respectively, were incubated with rabbit polyclonal Myoscape AB together with one of the following primary antibodies: monoclonal anti-α-Actinin (1:100: Sigma) or polyclonal anti-LTCC (1:100 Santa Cruz), at 4 °C overnight. Fluorescence labelling was carried out with secondary antibodies conjugated with goat anti-mouse fluorescein (1:200, Vector Laboboratries, Inc.) or goat anti-rabbit Cy3 (1: 200/Dianova) for 60 min at RT. Vectashield medium with DAPI (4',6'-diamidino-2-phenylindole; Vector Laboratories, Inc.) was used for mounting of the slides. Fluorescence micrographs were taken with Axioskop 2 Plus (Zeiss). Cell surface areas of cardiomyocytes were determined applying AxioVision Release 4.4 (Carl Zeiss Vision).

### Tissue culture experiments

HEK-293T cells (large ‘T' transformed embryonic kidney cells) were purchased from ATCC (293T-ATCC CRL-3216) and were maintained in DMEM medium supplemented with 10% FBS, 2 mM L-glutamine and penicillin/streptomycin under standard cell culture conditions (37 °C/5% CO_2_).

### Isolation and culture of adult rat ventricular cardiomyocytes (ARVCMs)

Adult rat cardiomyocytes (ARVCM) were isolated from Sprague–Dawley rats as described^59–60^. In brief, animals were anaesthetised with sodium pentobarbital (50 mg kg^−1^, intraperitoneal) and the aorta was rapidly cannulated after the hearts were excised and perfused with a rate of 10–12 ml min^−1^ in a Langendorff apparatus. Hearts were initially perfused with calcium-free AC medium (ACM) (pH 7.2) consisting of (in mM) 5.4 KCl, 3.5 MgSO_4_, 0.05 pyruvate, 20 NaHCO_3_, 11 glucose, 20 HEPES, 23.5 glutamate, 4.87 acetate, 10 EDTA, 0.5 phenol red, 15 butanedionemonoxime (BDM), 20 creatinine, 15 creatine phosphate (CrP), 15 taurine and 27 units per ml insulin under continuous equilibrium with 95% O_2_/5%CO_2_. After 5 min the perfusion was switched to ACM plus collagenase (0.5 U ml^−1^, type A; Roche diagnostics GmbH, Germany) for 20–30 min. Finally, perfusion was changed to low Na+, high sucrose Tyrode solution containing (in mM) 52.5 NaCl, 4.8 KCl, 1.19 KH_2_PO_4_, 1.2 MgSO_4_, 11.1.

Glucose, 145 sucrose, 10 taurine, 10 HEPES, 0.2 CaCl 2 for 15 min. Thereafter, left ventricles of digested hearts were cut into small pieces and subjected to gentle agitation to allow for dissociation of cells. Consequently, cells were resuspended in ACM without BDM in which 2 mM extracellular calcium ([Ca^2+^]) was gradually reintroduced at 25 °C. Cardiac myocytes to be used for contractility and Ca^2+^ measurements were plated with a density of 30,000 cells percm^2^ on laminin-coated dishes followed by adenoviral infection.

### Isolation and culture of NRVCMs

Hearts from 1-2 days old Wistar rats (Charles River) were excised and minced in ADS buffer (120 mmol l^−1^ NaCl, 20 mmol l^−1^ HEPES, 8 mmol l^−1^ NaH_2_PO_4_, 6 mmol l^−1^ glucose, 5 mmol l^−1^ KCl, 0.8 mmol l^−1^ MgSO_4_, pH 7.4). A series of digestion steps was carried out with an enzymatic solution containing collagenase type II (0.5 mg ml^−1^, Worthington) and pancreatin (0.6 mg ml^−1^, Sigma-Aldrich) in sterile ADS buffer. A Percoll (GE Healthcare) gradient centrifugation step was applied to remove contaminating fibroblasts from cardiomyocytes. NRVMs were resuspended and cultured in Dulbecco's modified Eagle's medium (DMEM) containing 10% FCS, penicillin/streptomycin and L-glutamine (PAA) for 24 h. After 24 h, cells were either infected in serum-free medium or serum starved for 24 h before applying stimulants or inhibitors for the indicated time^61^.

### Intracellular Ca2+ transients

Intracellular Ca^2+^ transients of Fura2-AM loaded ARVCMs (2 μmol l^−1^ for 20 min at 37 °C followed by 20 min incubation to allow for complete de-esterification of the dye) were obtained 24 h after plating and adenoviral infection as described above following a previously published protocol^59^,^60^. Measurements were carried out using an inverse Olympus microscope (IX70) with a UV filter connected to a monochromator (Polychrome II, T.I.L.L. Photonics GmbH, Germany). Cells were electrically stimulated with a biphasic pulse to contract at 37 °C at 2 Hz and excited at 340/380 nm. Epifluorescence emission was detected at 510 nm, digitized, and analysed off-line with T.I.L.L.VISION software (v. 3.3). Baseline data from 10 consecutive steady-state transients after 15 min of electrical stimulation were averaged for analysis of transient amplitudes. Isoproterenol (10-9-10-7M) was superfused through a gravity-fed perfusion system and Ca^2+^ transients were recorded 5 min after ßAR stimulation.

### Cardiomyocyte contractility

Contractile parameters in isolated ARVCM's were obtained by a blinded observer, 24 h after plating and adenoviral infection by video edge detection following a previously published protocol^57^,^58^. Briefly, contraction amplitudes were assessed in HEPES-modified medium 199. Analysis of steady-state twitches at 2 Hz field stimulation, 37 °C and 2 mM extracellular calcium concentration was performed by custom designed software written in LabView (version 5.0, National Instruments). Data from five consecutive steady-state twitches were averaged for analysis of fractional cellular shortening of ARVCM.

### L-type calcium current recordings

L-type calcium currents (ICa) were recorded in single cardiomyocytes with the whole-cell patch clamp configuration of the Multi Clamp 700 amplifier (Molecular Devices, Sunnyvale, CA, USA) as described below. Cardiomyocytes were incubated in external solution containing (mM): TEA-Br 140, CaCl2 2, Mg-acetate 1, Hepes 10, 4-amino-pyridine (4-AP) 5, CsBr 5, TTX 0.005, glucose 5, pH 7.4. Patch pipettes had tip resistances 3–6 MΩ when backfilled with internal solution of same composition. In initial experiments, cardiomyocytes were repolarized to various holding potentials, Vh, between −90 and −50 mV and ICa was elicited during 80-100 ms lasting depolarization's in 10 mV increments. ICa-V relations showed a prominent ‘shoulder' around −30 mV for Vh of −90 and −80 mV that arises from contributions of fast activating T-type currents. As these were completely eliminated for Vh of −50 mV, this holding potential was chosen for the remainder of the study. Apart from ICa activation protocols, steady-state inactivation was studied by applying double-pulse protocols (500 ms lasting pre-pulses to various potentials and a 150 ms lasting test pulse to 0 mV following a brief repolarisation to Vh and subsequent analysis. ICa-V plots, activation and inactivation curves were reconstructed from the data and half-activation d0.5 and half-inactivation F0.5 values extracted from least-square Boltzmann fits.

### Experimental rat heart failure model

All animal procedures and experiments were performed in accordance with corresponding institutional guidelines (Regierungspräsidium Kiel. Germany). Heart failure (HF) in 10–12 weeks old Sprague–Dawley rats of either sex was induced as described below, following an approved model of left ventricular (LV) cryoinfarction^26^,^28^. Briefly, rats were sedated with pentobarbital (65 mg kg^−1^) by intraperitoneal injection, intubated and anaesthesia under mechanical ventilation (Hugo Sachs, Germany) was maintained using 2% isoflurane (v/v) supplemented with oxygen. Subsequently, the heart was exposed through a median sternotomy and a 6-O suture was placed at the apex of the LV to stabilize the hearts during cryothermia. Cryothermia was applied by the use of liquid N2 cooled cylindrical probe (Ø 8 mm). In total, the probe was applied three times for 1 min to the LV free wall, and each cycle was interrupted by a 1-min thawing interval resulting in a transmural myocardial necrosis. Then, the chest was closed and animals were transferred back to their cages receiving appropriate analgesia due to an approved protocol. Animals were kept under constant observation for a period of 8h after surgery and inspected twice a day for a period of 1 week post intervention. Heart failure was assessed by systolic dysfunction in echocardiographic recordings. Sham-operated control animals underwent a similar procedure except application of the cryoprobe.

### Plasma membrane protein biotinylation assay

To purify plasma membrane proteins neonatal rat cardiomyocytes were plated for 16 h and infected with AdMiMyoscape vs AdMiNeg (100 i.f.u. each) and AdMyoscape vs AdLacz (50 i.f.u. each,) respectively, for at least another 24 h. The plasma membrane proteins were labelled with cleavable biotinylation reagent (Sulfo-NHS-SS-Biotin) and purified with Neutravidin-Agarose-Resin according to the manufacturer's instructions (Pierce Cell Surface Protein Isolation Kit). Purified proteins were compared against total cellular protein-components. Equal loading was confirmed via protein amount measurements and additional Ponceau staining.

### Zebrafish morpholino-mediated knock-down

Care and breeding of zebrafish *Danio rerio* was done under standardized and established conditions^60^,^61^. The present study was performed under institutional approval, which comply by the Guide for the Care and Use of Laboratory Animals published by the US National Institute of Health (NIHPublication No. 85–23). Morpholino-modified oligonucleotides were directed against the splice donor site of intron 2 (MO-Myoscape=5′-TTGTTTTTGTTAGTGTCTGACCTGA-3′) of zebrafish Myoscape. A standard control oligonucleotide (MO-control; GENETOOLS, LLC) was injected at the same concentration as a negative control^62^,^63^,^64^. To inhibit pigmentation, 0.003 % 1-phenyl-2-thiourea was added to the embryo medium. Pictures and movies were recorded 24, 48 and 72 h after fertilization.

### Zebrafish and mouse heart histology and immunostaining

Zebrafish embryos, mouse hearts and skeletal^5^ muscle sections were fixed in 4% paraformaldehyde and embedded in JB-4 (Polysciences, Inc.). 5 μm sections were cut, dried, and stained with hematoxylin/eosin. For transmission electron microscopy, zebrafish embryos were fixed overnight in 3% glutaraldehyde in 100 mM cacodylic acid (pH 7.4) supplemented with 0.1% picrinic acid at 4 °C, they were treated with OsO4 and dehydrated in a graded series of ethanol. Epon 812 was used for embedding. Subsequently, ultrathin sections (70 nm) were obtained with an Ultracut E microtome (Leica), stained with uranyl acetate and Reynolds lead citrate and examined on a Philips EM 301 transmission electron microscope. For whole-mount double immunostaining, embryos were fixed in Dent's fixative and stained with monoclonal antibodies directed against atrial and ventricular meromyosin (MF20; DSHB; working dilution 1:100) and against the atrial specific isoform of myosin heavy chain (S46; DSHB; working dilution 1:100).

### Functional assessment of zebrafish morphants

Still images and video films were recorded and digitized with a Zeiss MCU II microscope connected to a digital camera. Functional assessment of cardiac contractility was carried out at the indicated time points post fertilization^60^,^61^. Ventricular fractional shortening fraction was calculated from the differences of maximum diastole and maximum systole measurements with the help of the zebraFS software (http://www.benegfx.de).

### Generation of Myoscape KO mice

Constitutive Myoscape KO mice were generated in cooperation with Tacronic – Artemis. The corresponding Gene Identifier Accession was ENSMUSG00000039629. The general targeting strategy allowed generation of conditional and constitutive Myoscape KO (Supplementary Fig. 12) First the Myoscape exons 3–8 were flanked by loxP sites. The desired Neo selection marker was flanked by FRT sites, and Puro by F3 sites. This strategy allowed either a generation of a Myoscape-Conditional KO after *in vivo* Flp-mediated removal of this selection marker and a Constitutive KO by Cre-mediated deletion of exons 3–8. The estimated deletion of exons 3–8 should result in loss of function by deletion of the N1221 like protein domain and generation of a frameshift.

### Vector construction

Mouse genomic fragments were subcloned using a RP23 BAC library and cloned into the basic targeting vector harbouring the indicated features. If necessary, additional fragments were amplified by PCR and subcloned.

### ES cell culture (B6)

The C57BL/6N ES cell line (TaconicArtemis C57BL/6N *Tac ES* cell line) was grown on a mitotically inactivated feeder layer comprised of mouse embryonic fibroblasts (MEF) in DMEM High Glucose medium containing 20% FBS (PAN) and 1,200 μ ml^−1^ Leukaemia Inhibitory Factor (Millipore ESG 1107). 1 × 107 cells and 30 g of linearized DNA vector were electroporated (Biorad Gene Pulser) at 240 V and 500 F. Puromycin selection (1 g ml^−1^) and G418 selection (200 g ml^−1^) was started on day 2. Counterselection with Gancyclovir (2 M) started on d5 after electroporation. ES clones were isolated on d8 and analysed by Southern Blotting according to standard procedures after expansion and freezing of clones in liquid nitrogen.

### Transfection of ES cells

Transfection of ES cells was carried out by electroporation of the designed Vector pCamp1 Final LA_5.2 into the ES cell line C57BL/6N. The selection method used G418 resistance, Puromycin resistance and a counterselection with Gancyclovir. A number of 252 ES Clones were screened. The screening strategy was done by repeated southern analysis where 16 successfully targeted clones could be identified. A cell quality control was done by a mycoplasma test.

### Diploid blastocyst injection

After administration of hormones, superovulated Balb/c females were mated with Balb/c males. Blastocysts were isolated from the uterus at dpc 3.5. For microinjection, blastocysts were placed in a drop of DMEM with 15% FCS under mineral oil. A flat tip, piezo actuated microinjection-pipette with an internal diameter of 12–15 μm was used to inject 10–15 targeted C57BL/6N.tac ES cells into each blastocyst. After recovery, eight injected blastocysts were transferred to each uterine horn of 2.5 days post coitum, pseudopregnant NMRI females. Chimerism was measured in chimeras (G0) by coat colour contribution of ES cells to the Balb/c host (black/white). Highly chimeric mice were bred to strain C57BL/6 females. Depending on the project requirements, the C57BL/6 mating partners are non-mutant (W) or mutant for the presence of a recombinase gene (Flp-Deleter or Cre-deleter or CreER inducible deleter or combination of Flp-deleter/CreER). Germline transmission was identified by the presence of black, strain C57BL/6, offspring (G1). Genotyping was carried out using primers and protocols shown in Supplementary Table 1.

### Echocardiography and phenotyping of mice

Mice were briefly anaesthetized with isoflurane (Havard-apparatus). Echocardiographic measurements were performed by a blinded observer using a GE Vivid 7 ultrasound machine. Animals were sacrificed and hearts, lungs and liver weights were recorded. Heart samples were collected for RNA and protein isolations and were embedded in OCT for histological analyses. Six lead limb surface echocardiogram were assessed in male and female WT and transgenic mice under brief anaesthesia with isoflurane. Heart rates, QT and QTc duration intervals were analysed by semi-automated shape analysis and measurements. Blood pressure was measured non-invasively.

### *In vivo* electrophysiological experiments in mice

Ventricular cardiomyocytes were isolated as described (AfCS Procedure Protocol PP00000125), maintained at 37 °C, and aerated with 2% CO_2_.The mouse was first injected intrapertoneally (i.p.) with 0.5 cc heparin diluted in phosphate buffered saline (PBS) to 100 IU ml^−1^ followed by anaesthesia with ketamin 100 mg ml^−1^, xylazine (Rompun) 2%, acepromazin (Vetranquil) 1% in PBS i.p. In brief, perfusion buffer (113 mM NaCl, 4.7 mm KCl, 0.6 mM KH_2_PO_4,_ 0.6 mM Na_2_HPO_4_, 1.2 mM MgSO_4_-7H_2_0, 0.032 mM Phenol red, 12 mM NaHCO_3_, 10 mM KHCO_3,_ 10 mM HEPES Buffer 1 M, 30 mM Taurine, 5 mM Glucose, 10 mm 2,3-Butanedione-monoxime) and digestion buffer (perfusion buffer with 0.25 mg ml^−1^ Liperase blendzyme 1 (Roche), 0.14 mg ml^−1^ Trypsin (GIBCO), 12,5 μM CaCl_2_) were warmed to 37 °C before use. The flow rate of the pump was adjusted to 3 ml min^−1^ and the perfusion system was equilibrated with water and perfusion buffer for at least 5 min. After anaesthesia the chests of mice were wiped the chest with 70% ethanol and the peritoneal cavity and chest was opened with small scissors. The heart was removed from extracardiac tissue and was cannulated to the perfusion apparatus. The perfusion was started immediately (3 ml min^−1^) with perfusion buffer for 4-5 min. Then the perfusion was switched to the digestion buffer and perfused for 8–10 min at 3 ml min^−1^. after enzyme digestion of the heart was completed, the ventricles were cut into several small pieces with fine forceps. The resulting cell suspension was rinsed with 2.5 ml of room temperature myocyte stopping buffer 1 (perfusion buffer with 10% BCS, 12.5 μM CaCl_2_). For Calcium reintroduction the myocyte pellet was suspended in room temperature myocyte stopping buffer 2 (perfusion buffer with 5% BCS, 12.5 μM CaCl_2_) to a total volume of 10 ml. Stepwise, increasing amounts of calcium chloride, (10 mM CaCl_2_) was added and at each step, the suspension was incubated for 4 min at room temperature. Finally, the myocytes were transferred to a new 15-ml tube and were allowed to sediment by gravity (8–10 min). The resulting pellets were suspended in 5 ml of MC plating medium (Minimum essential medium (MEM), 1 × (with Hanks' salts and L-glutamine, 5% BCS, 10mM BDM, 5 U ml^−1^ Penicillin, 2 mM L-Glutamine) at 37 °C. After counting viable cells, myocytes were then plated to laminin-pre coated dishes.

### Electrophysiological recordings

Whole-cell Ca^2+^ was measured at room temperature. Stimulation and data acquisition were performed by Multi Clamp 700 A and pClamp 9.0 Software (Axon Instruments, Foster City, USA). Data were sampled at 5–20 kHz and filtered at 1 kHz. The input resistance of the patch pipettes ranged from 1 to 1.3 MΩ when filled with intracellular solution. Extracellular solution was composed of (in mM): NaCl 137, CaCl_2_ 1.8 (BaCl_2_ 1.8 for measuring CDI), CsCl 25, MgCl_2_ 0.5, Glucose 10, HEPES 10, pH 7.4 adjusted with NaOH. Intracellular solution was composed of (in mM): CsCl 120, TEA-Cl 10, EGTA 1, MgATP 1, Na_2_GTP 1, HEPES 10, phosphocreatine 5, pH 7.2 adjusted with CsOH. To measure current–voltage (I–V) relationships, CMs were stimulated by the voltage protocol (A). A prepulse from −80 to −40 mV was used to inactivate fast Na^+^ currents. Current traces and voltage protocols are superimposed from −40 to +60 mV. Current–voltage relation of *I*_Ca_. Peak current density is plotted against the voltage pulse.


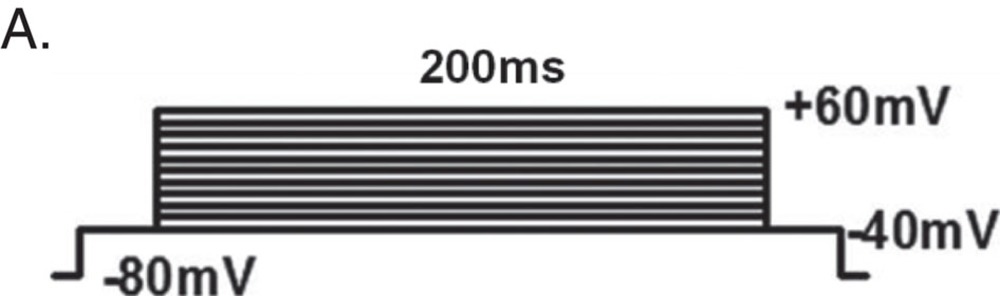


Facilitation of ICa was measured in CMs stimulated by a twin-pulse protocol (B).


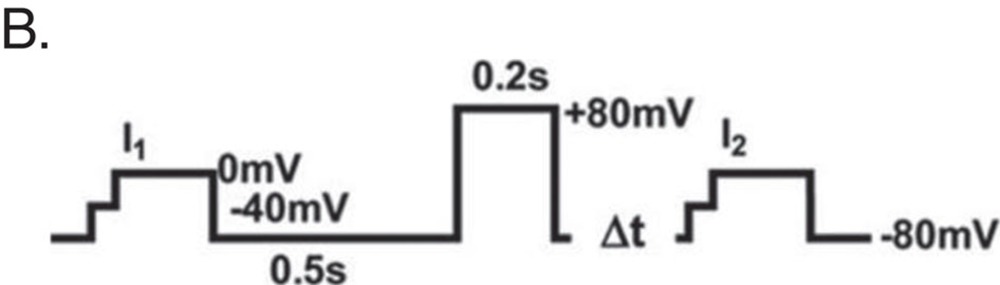


Calcium-dependent inactivation was measured using the IV protocol. After adjusting the baseline, peak amplitude was normalized. Time constant was calculated after fitting the data sets with two exponential functions.

For measuring steady-state inactivation CMs were stimulated by a twin-pulse protocol (C). Fractions of current (*I*_2_/*I*_1_) were plotted against the prepulse voltage. *E*_0.5_ was calculated after fitting the data sets with a Boltzmann equation.


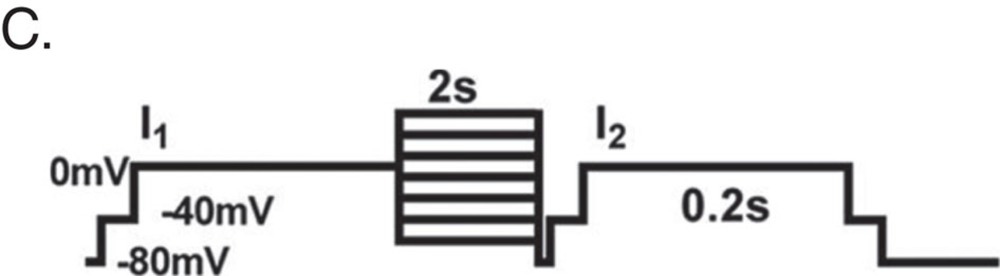


INa was inhibited in every experiment by a 30-ms depolarization to −40 mV preceding the test pulse. Data plotting and statistical analysis was carried out using GraphPad PRISM 5.0 (GraphPad Software, Inc., La Jolla, USA).

### TAC in mice

TAC was performed in male C57BL6 mice (8–10 weeks old). Briefly the animals were anaesthetised with ketamine (120 mg kg^−1^ i.p.) plus xylazine (15 mg kg^−1^ i.p.). The mice were orally intubated with 20-gauge tubing and ventilated (Harvard Apparatus) at 120 breaths per minute (0.2 ml tidal volume). The aortic constriction was created via a lateral thoracotomy at the second intercostal space. A suture (Prolene 6-0) was placed around the transverse aorta between the brachiocephalic and left carotid artery. The suture was ligated against a 27-gauge needle, the needle was subsequently removed leaving a discrete stenosis. The chest was closed and a pneumothorax evacuated. Sham animals underwent the same the procedure except ligation.

### Cross sectional area measurements

Hearts were transversely sectioned at 7 μm thickness and fixed with 4% paraformaldehyde, and permeabilized with Triton X. Cryoslices were incubated with a mouse monoclonal anti-vinculin antibody (Sigma-Aldrich) for 2 h. Cells were then incubated with a Alexa Fluor488 coupled goat anti-mouse secondary antibody (Molecular Probes). Images of stained cardiomyocytes were acquired on Zeiss Observer Z.1 microscope (Carl Zeiss). The outlines of a least 300 cardiac myocytes were traced by using Axio Vision software (Carl Zeiss).

### Sirius red staining

For Sirius red staining heart cryosections after TAC and sham procedures were dried for 1 h. The staining was started with incubation for 4 days with 1.2% Pikrin acid (Applichem GmbH #A2520) with 0.2% Fast Green FCF (Merck #1.04022.0025) and 0.1% Sirius Red (= Direct Red 80; Sigma-Aldrich #365548). Then sections were dried in different increasing alcoholic dilutions.

### Statistical analyses

All results are shown as the mean±s.e.m. unless stated otherwise. Statistical analyses of the data were carried out using one-way ANOVA followed by Student–Newman–Keuls *post hoc* tests. If appropriate, Student's *t*-test (two sided) was employed. *P* values <0.05 were considered statistically significant. The ΔΔct method was used to analyze qPCR^57^,^58^.

## Additional information

**How to cite this article:** Eden, M. *et al*. Myoscape controls cardiac calcium cycling and contractility via regulation of L-type calcium channel surface expression. *Nat. Commun.* 7:11317 doi: 10.1038/ncomms11317 (2016).

## Supplementary Material

Supplementary Information Supplementary Figures 1-12, Supplementary Table 1, Supplementary Note 1 and Supplementary References

Supplementary Movie 1 Supplementary movie 1 shows video microscopy loops of impaired heart function from Myoscape morphant zebrafish at 48hpf.

Supplementary Movie 2 Supplementary movie 2 shows video microscopy of control morpholino infected zebrafish hearts at 48hpf
