# Erratum: Myoscape controls cardiac calcium cycling and contractility via regulation of L-type calcium channel surface expression

**DOI:** 10.1038/ncomms11835

**Published:** 2016-05-26

**Authors:** Matthias Eden, Benjamin Meder, Mirko Völkers, Montatip Poomvanicha, Katrin Domes, M. Branchereau, P. Marck, Rainer Will, Alexander Bernt, Ashraf Rangrez, Matthias Busch, Martin Hrabě de Angelis, Christophe Heymes, Wolfgang Rottbauer, Patrick Most, Franz Hofmann, Norbert Frey

Nature Communications 7, Article number: 11317 10.1038/ncomms11317 (2016); Published: 04282016; Updated: 05262016

The original version of this Article contained an error in the doi number, which was incorrectly given as 15.13155/ncomms11317. This has now been corrected in both the PDF and HTML versions of the Article.

