# Supplementary material for: Myoscape controls cardiac calcium cycling and contractility via regulation of L-type calcium channel surface expression
Source: Nat Commun. 2016 Apr 28;7:11317. doi: 10.1038/ncomms11317 (PMC5438100; doi:10.1038/ncomms11317)
Supplement: Supplementary Information — Supplementary Figures 1-12, Supplementary Table 1, Supplementary Note 1 and Supplementary References [file ncomms11317-s1.pdf]

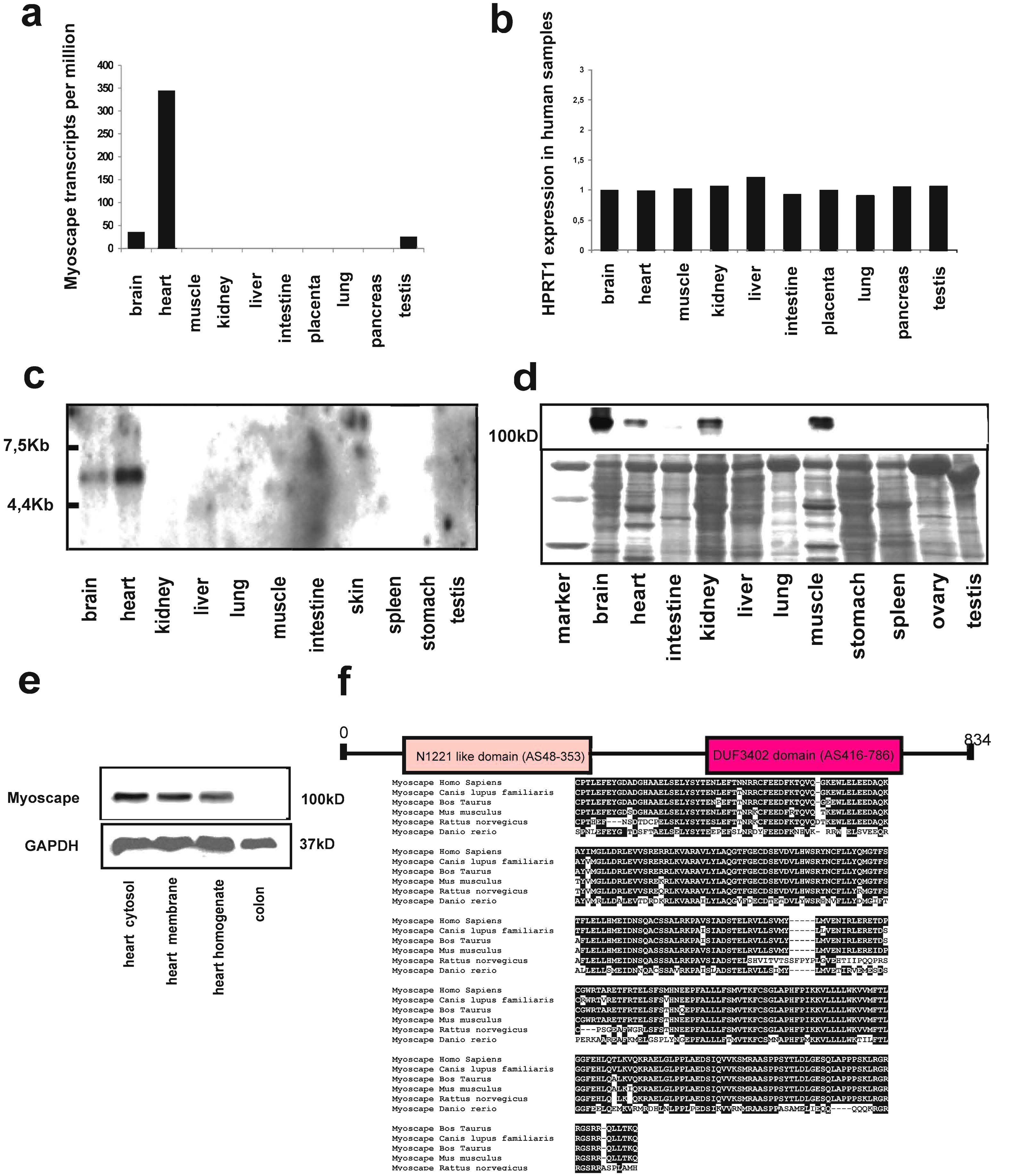

Supplementary Figure 1

(a) Mouse EST database-predicted muscle- and heart-enriched mRNA-expression profile. **(b)** Confirmation of stable and equal expression of HPRT1 mRNA by realtime PCR presented by n-fold expression vs. brain tissue. EST profile was additionally confirmed by northern-blot analyses of mouse tissues using murine Myoscape-specific probes and primers **(c)**. **(d)** Probing an equally loaded, ready to use INSTA Blot - multiple human tissue western blot - membrane (Novusbio) with an anti-Myoscape antibody (SIGMA) also shows a significant protein abundance in heart- and muscle. Bottom shows amido black staining by the manufacturer. However, in humans Myoscape shows also a strong abundance in brain and kidney. **(e)** Analysis of cellular subfractions using anti-Myoscape antibody (SIGMA) revealed that Myoscape protein is equally detectable in cytosolic as well as in membrane fractions at the expected size of 105 kDa. **(f)** Protein sequence alignment between human (NP_065755.1), mouse (NP_796178.2), rat (rCG27982), chimp (XP_519374.3), dog (XP_8490003.1), bovine (XP_003582169.1), and zebrafish (CAKO4721) Myoscape shows high conservation among species, as indicated by the black bars above the calculated consensus sequence.

a

Myoscape Epitope:

-PPLAEDSIQVVKSMRAASPPSYTLDLGESQLAPPPSKLRGRRGSRRQLLTKQDSLDIYNE-

Peptide Sequence **H₂N SK LRG RRG SRR QLL T CONH₂**

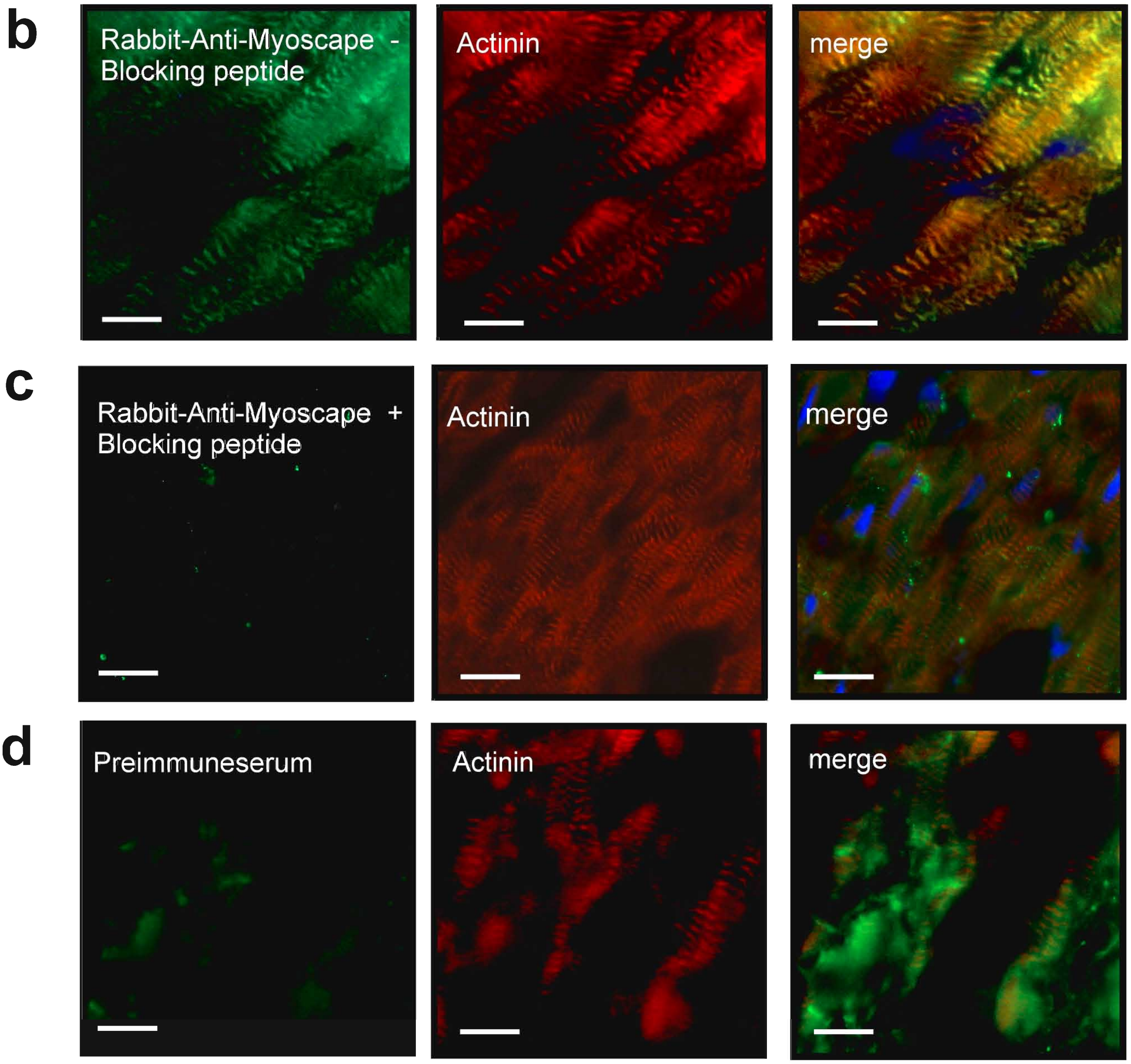

Supplementary Figure 2

(a) Immunohistological evaluation of our polyclonal anti-Myoscape antibody produced against the presented Myoscape epitope sequence in rabbits (Eurogentech). **(b)** While staining with this anti-Myoscape-antibody showed a strong striated signal colocalizing with α-Actinin, **(c)** co-incubation with the specific Myoscape recognition/blocking peptide against AB epitopes abolished the z-band-specific detection signal in mouse heart tissues. **(d)** Serum samples of rabbits prior to immunization did not produce a specific immunofluorescent signal.

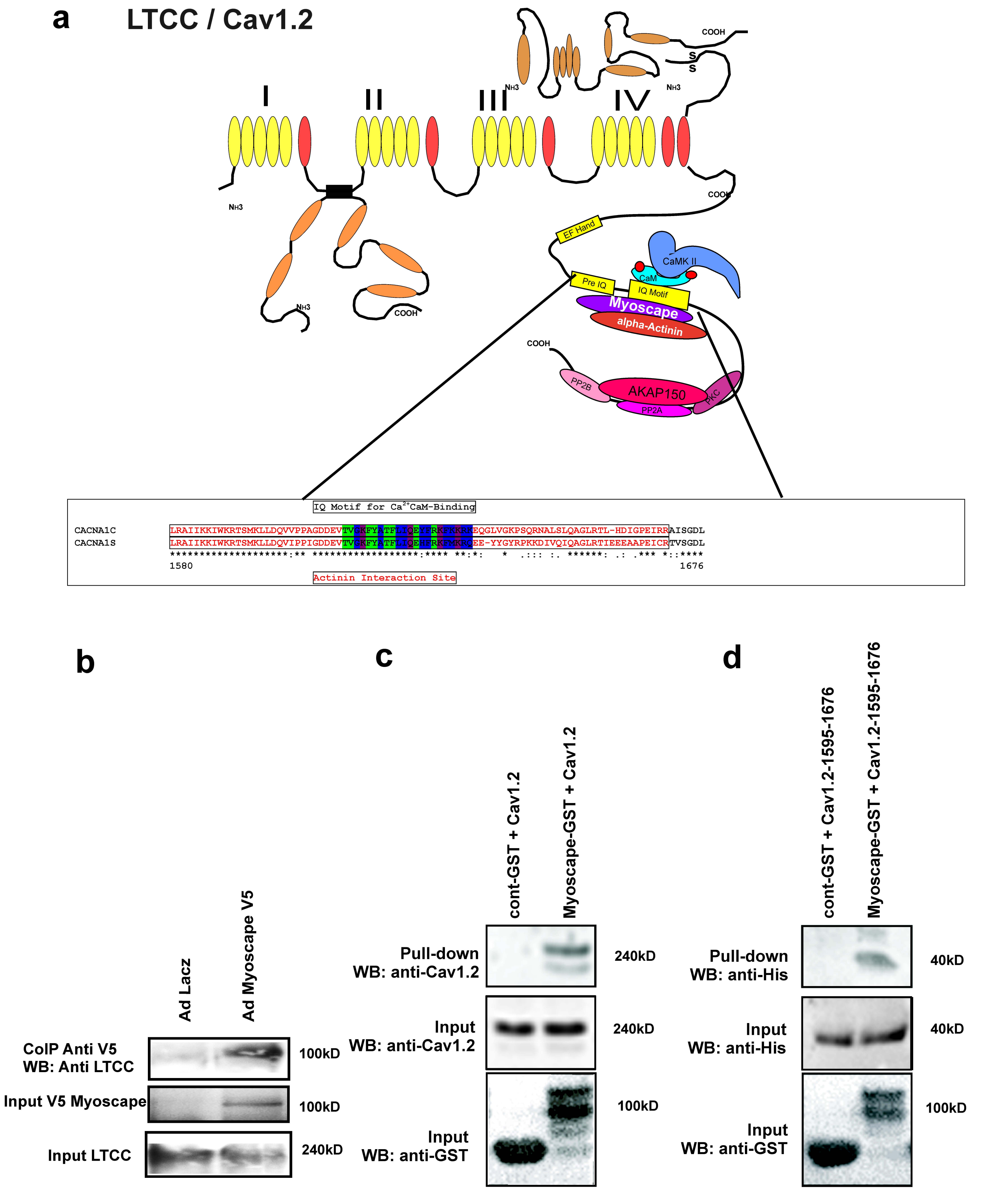

Supplementary Figure 3

(a) Theoretical model showing the interaction domain of LTCC distal terminal tail and in relation to α-Actinin and other known regulatory interacting proteins and phosphorylation sites. (b). Confirmation of the interaction between the cardiac specific isoform of the endogenous CACNA1C protein and adenoviral overexpressed Myoscape-V5 tagged-protein by co-immunoprecipitation of the proteins in NRVCM. GST pull-down assay performed between Myoscape and either full-length (c), or C-terminal binding domain of the LTCC/Cav1.2 (1595-1676) derived from Yeast-two hybrid assay (d) indicating the direct interaction of Myoscape with amino acid sequence 1595-1676 of the C-terminus of Cav1.2.

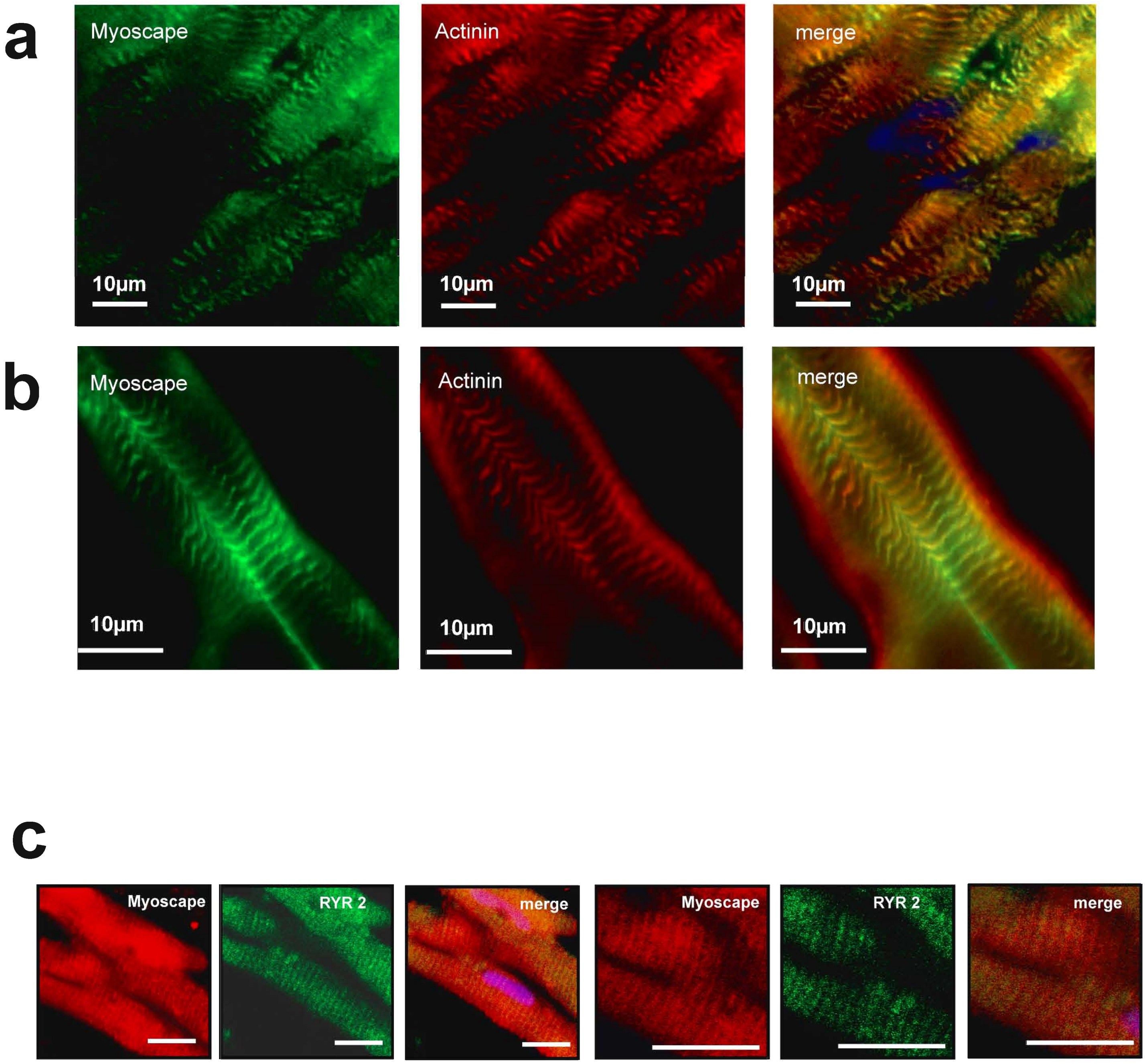

Supplementary Figure 4

Consistent with the localization in adult mouse heart **(a)**, staining of intact mouse skeletal muscle **(b)** cryosections with Myoscape showed colocalization with α-Actinin at the z-band t-tubule interface. Scale bar 10µm **(c)** By additional confocal coimmunostaining of Myoscape and RYR2 in ARVCM, Myoscape could also be localized to couplons near the sarcomeric z-band/t-tubule interface. Scale bar 20µm

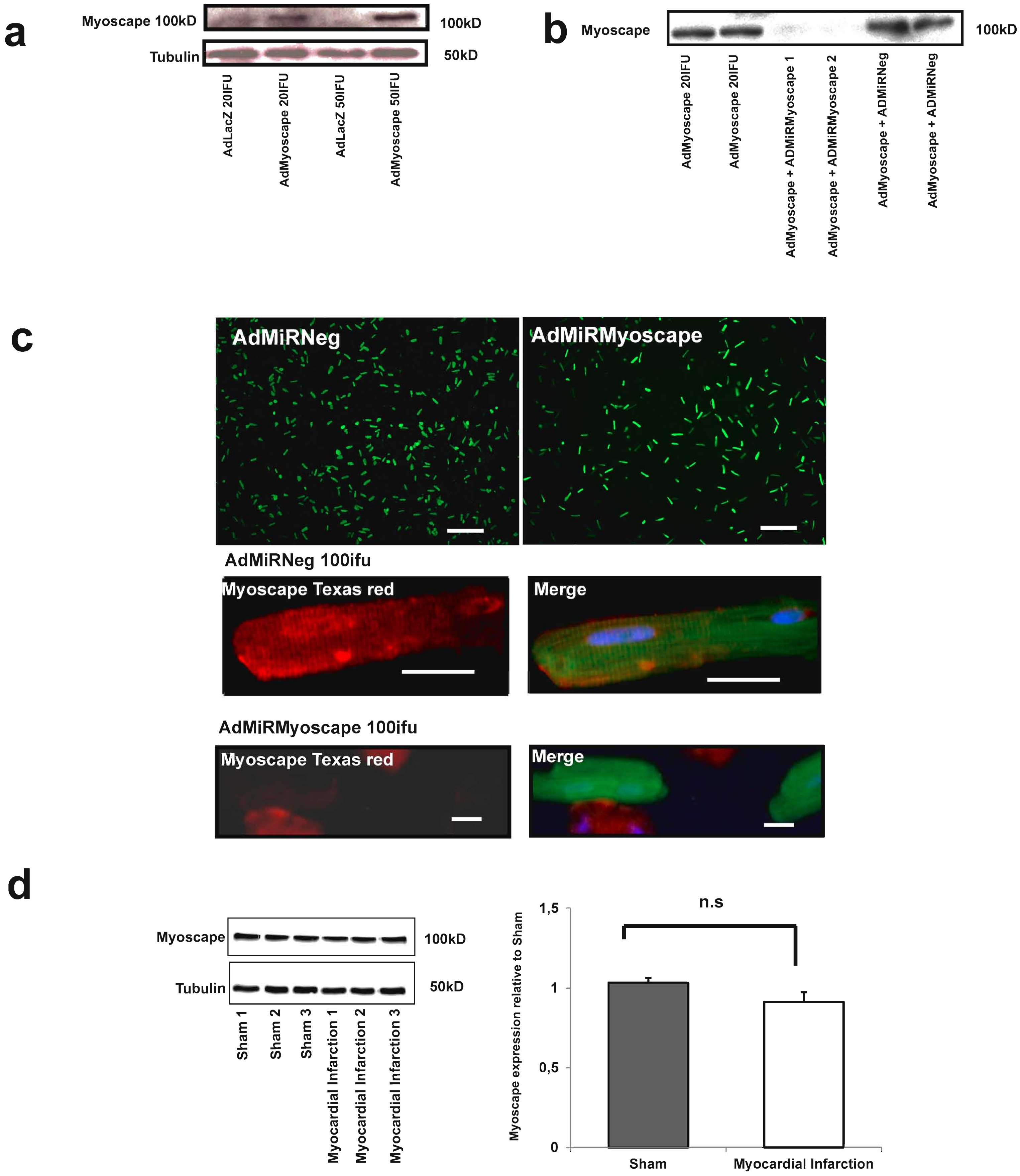

Supplementary Figure 5

(a) Western blot experiments showing a clear induction of Myoscape protein content in ARVCM after 24 h of infection with 20 ifu of AdMyoscape compared to AdLacZ. **(b)** Western blot experiments showing a clear and almost complete ablation of even adenoviral overexpressed Myoscape protein (20 ifu AdMyoscape) in ARVCM after 24 h of infection with 100 ifu of AdMiRMyoscape compared to AdMiRNeg as control. **(c)** Immunohistological experiments showed that after 24 h of infection with 100 ifu of GFP labeled AdMiRMyoscape compared to GFP labelled AdMiRNeg, a strong viral transduction was observed (Scale bar 200µm). This resulted in >90% GFP positive ARVCM's as well as specific MiRNA-infected cells did not express detectable Myoscape protein, whereas MiRNeg-infected cells still showed striated Myoscape expression (Texas red Scale bar 20µm). Western blot experiments in heart tissue of sham operated or LAD ligated pig hearts, show no differential regulation of Myoscape protein in short term cardiac failure **(d)**.

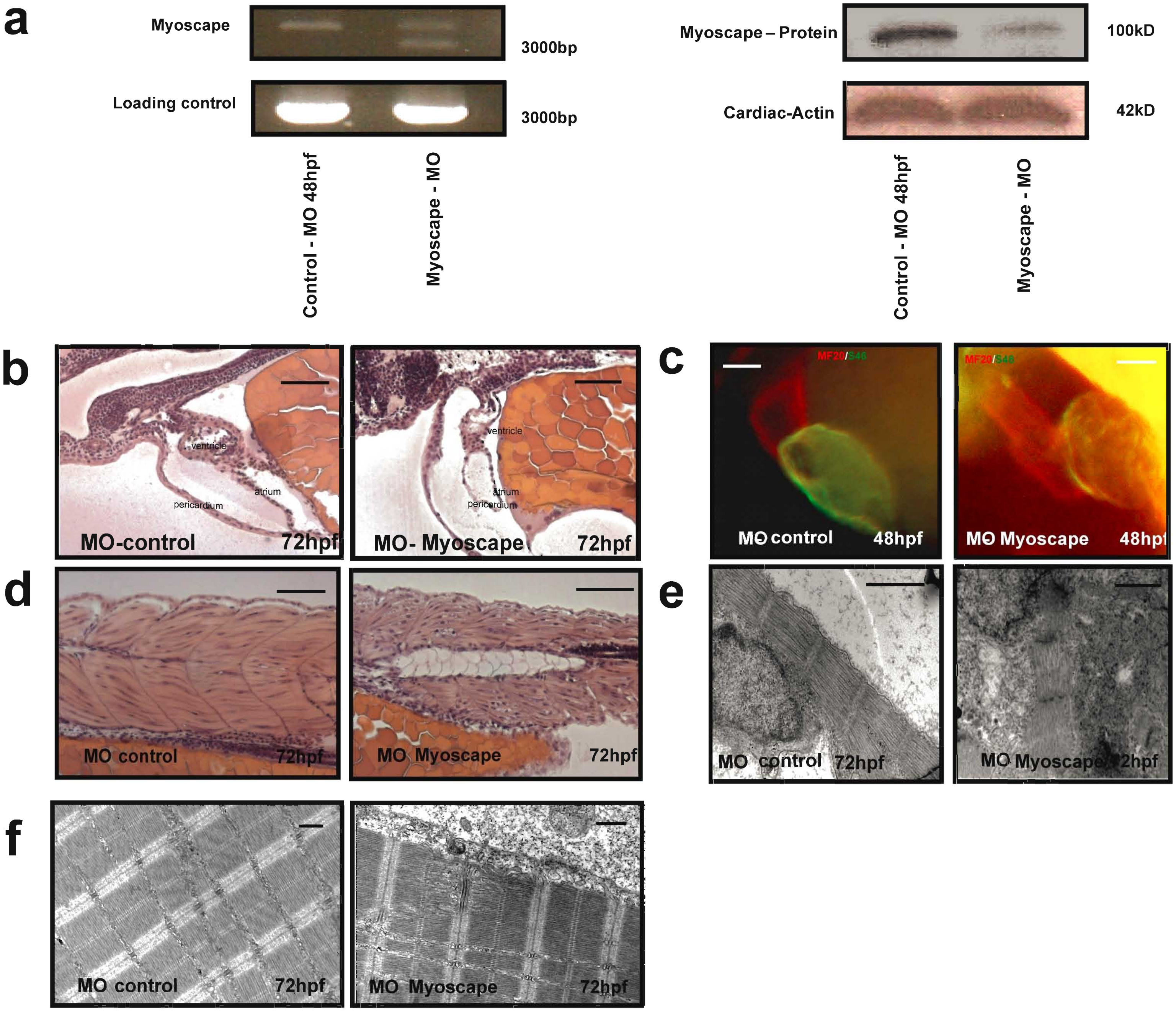

Supplementary Figure 6

(a left panel) RT-PCR analysis in zebrafish hearts from Myoscape knockdown morphants compared to controls showed a successful splice site mutation as indicated by a clear double band. **(a right panel)** Western blot analysis of zebrafish hearts from Myoscape knockdown morphants compared to controls, revealing a significant (>60%) reduction of Myoscape protein content after morpholino injection. **(b+d)** Histological (HE staining, scale bar 100µm), immunohistochemical (scale bar 100µm) **(c)** and ultrastructural evaluation of zebrafish heart **(e)** sections and skeletal muscle tissue **(f)** showing no gross architectural deficits after Myoscape ablation, indicating no obvious role of Myoscape in development or cellular sub-architecture of these tissues in zebrafish (scale bar EM 1µm).

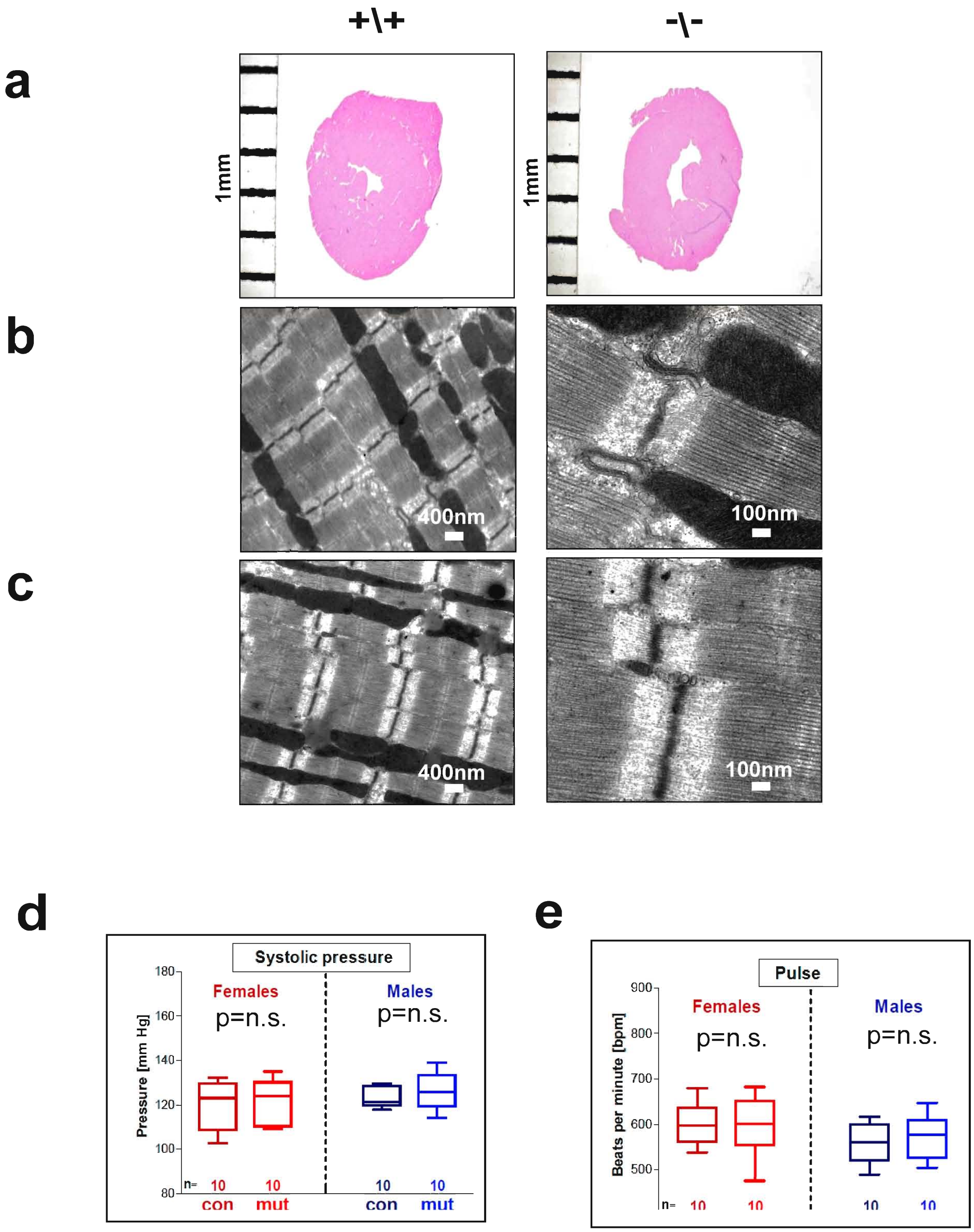

Supplemenaryl Figure 7

(a) Histological (HE staining) evaluation of WT and KO mice heart sections again showing no gross architectural deficits and no severe cardiac hypertrophy in the absence of Myoscape protein. Electron microscopy of **(b)** heart and **(c)** skeletal muscle tissues of wild type (+/+) and Myoscape knockout mice (-/-) again revealed no gross architectural defects regarding either sarcomeric structures or the z-disc t-tubule interface /dyad/triad). The width of the z-discs and the size, length, and amount of t-tubules appeared normal. **(d and e)** Genetic ablation of Myoscape does not alter blood pressure or heart rates in vivo.

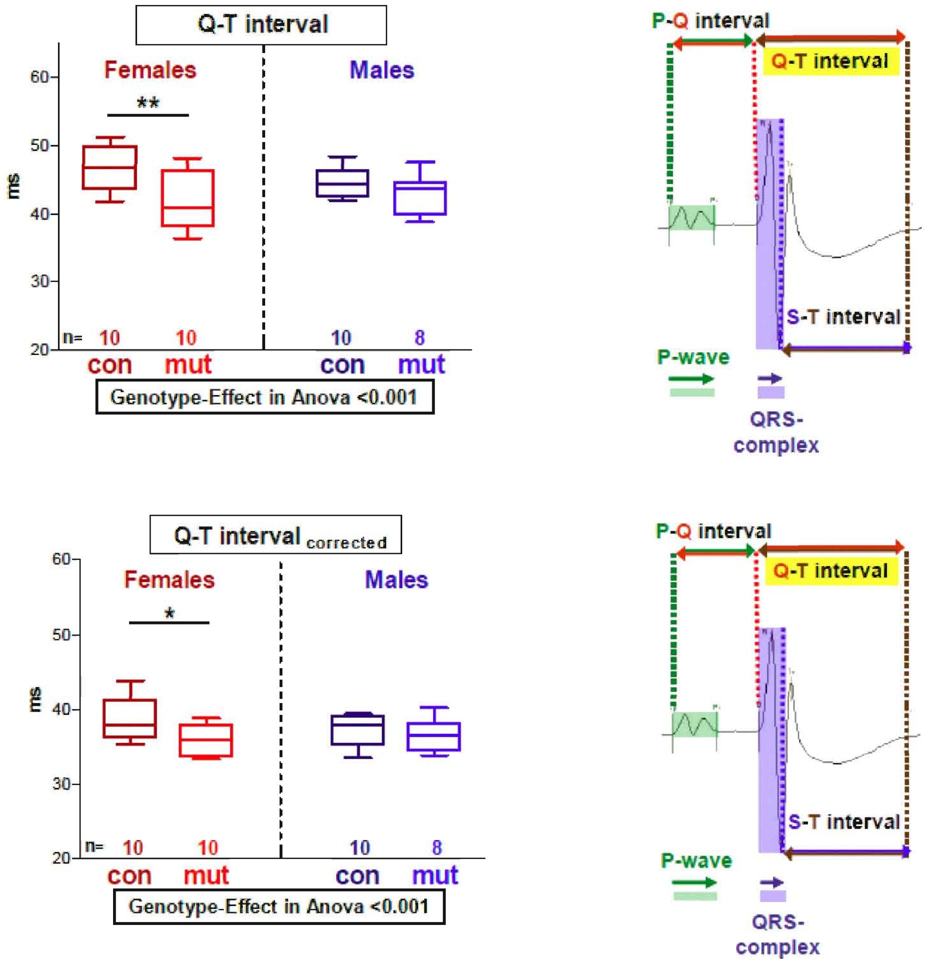

Supplementary Figure 8

A 6 lead limb surface ECG was performed in n=8-10 female and male Myoscape wildtype and Myoscape knockout mice. Satistical analysis (ANOVA) showed a significant shortening of the Qt interval (upper panel) and the corrected Qtc duration (lower panel) in female Myoscape null mice. p<0,001. Whereas in males only a strong trend was observed which not reached statistical significance

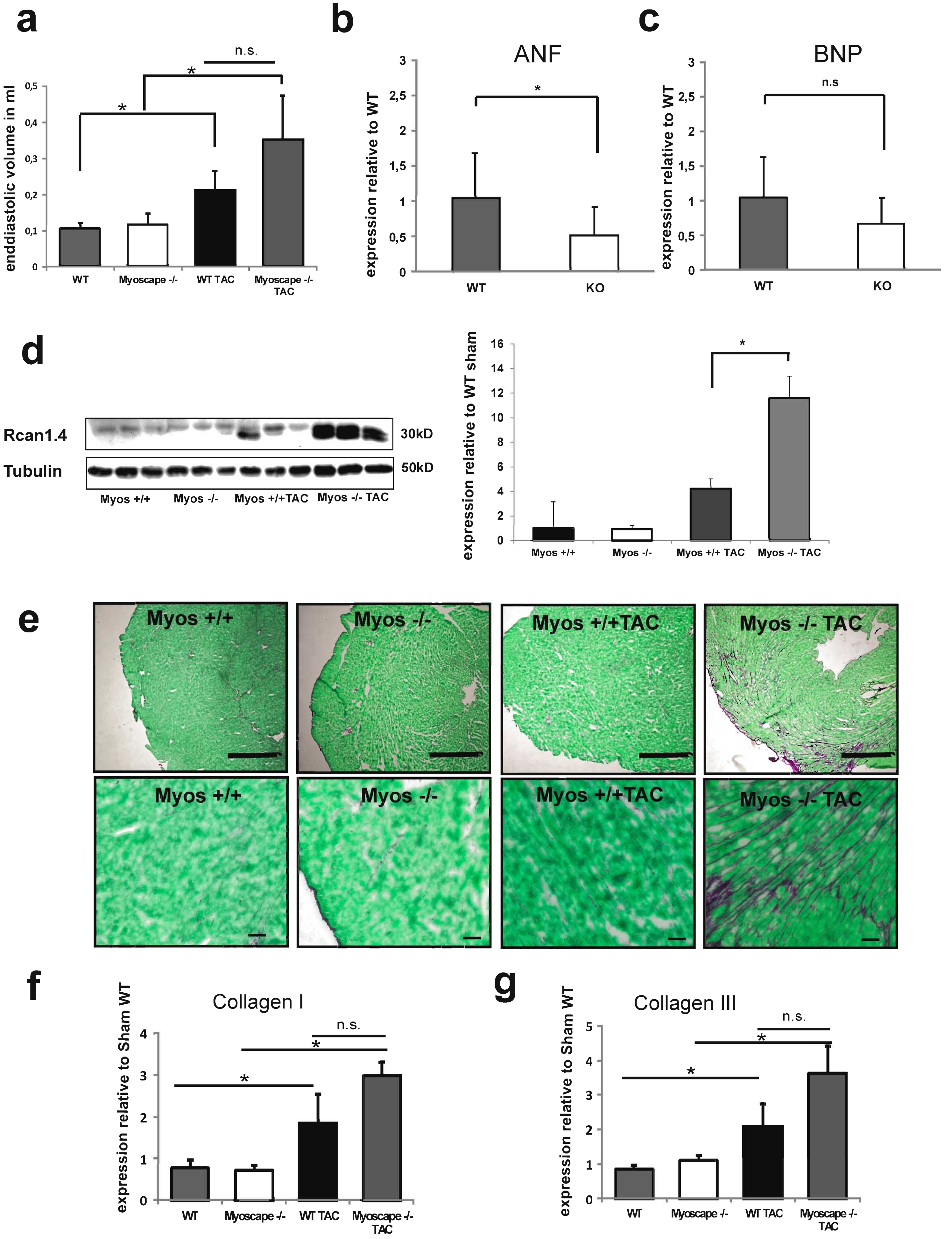

Supplementary Figure 9

(a) Invasive measurements of LV volumes in WT and KO mice under basal conditions and after TAC showed increased LV volumes in all mice after aortic constriction, with a strong but not significant trend to even larger ventricles in Myoscape KO hearts. **(b)** QPCR analyses of baseline WT and KO mice showed a slightly but significantly reduced ANF expression. In contrast, BNP mRNA levels did not significantly differ between WT and KO littermates, consistent with the lack of severe hypertrophy observed in our mice under unchallenged conditions. **(c)** Western blot analysis in WT and KO animals after TAC or sham surgery revealed a strong increase of Calcineurin activity assessed by RCAN1.4 induction **(d)**. Moreover, sirius red staining of heart sections after TAC as well as QPCR analysis of collagen I and II mRNA synthesis in WT and KO mice after aortic constriction showing a trend toward increased fibrosis in response to biomechanical stress in the absence of Myoscape **(d and e)**. * $p<0.05$. Scale bar 500µm upper panel and 50µm bottom

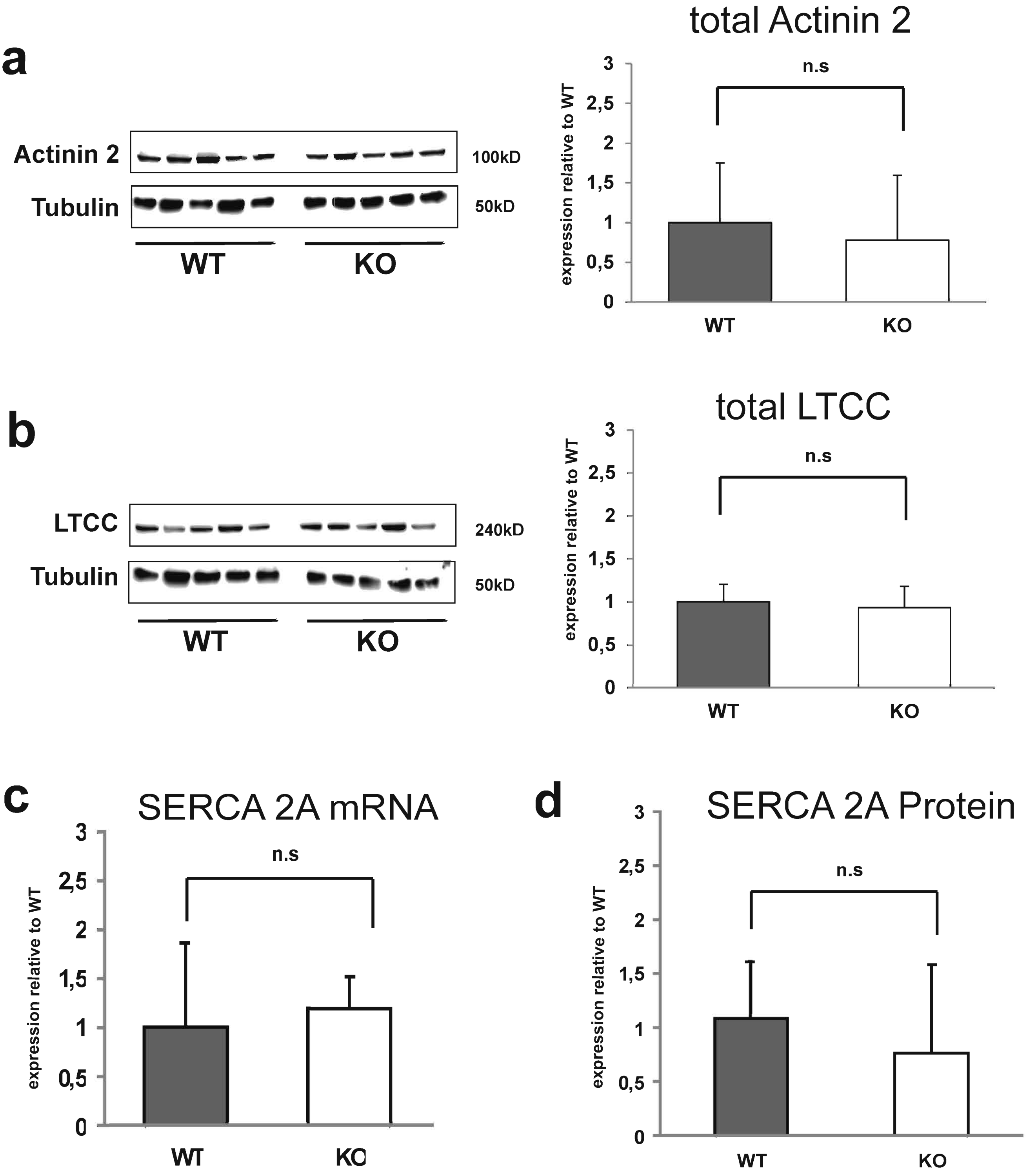

Supplementary Figure 10

(a) Western blot analyses of cardiac α-Actinin 2 content in WT and KO mice showed no significant differences in the absence of Myoscape. **(b)** Consistently, total heart LTCC levels did not significantly differ between WT and KO littermates, consistent with the notion that Myoscape does not modulate overall LTCC expression on a transcriptional level, whereas it regulates membrane retention of previously synthesized channels. **(c)** QPCR analyses of WT and KO mice SERCA 2A mRNA and **(d)** western blot analyses showing SERCA 2A protein content could not reveal differential regulation.

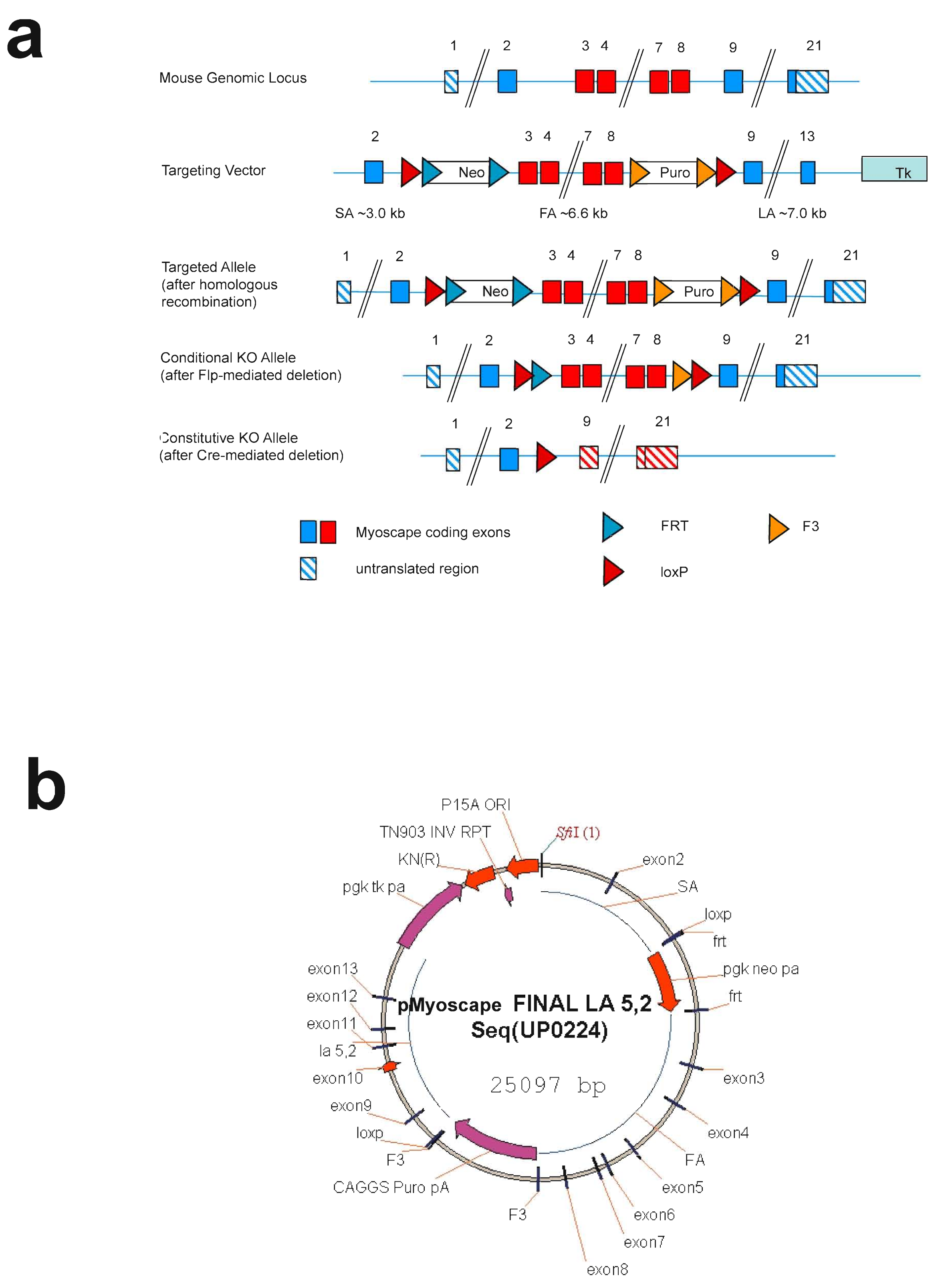

Supplementary Figure 11.
(a) Schematic KO strategy. This strategy allowed either a generation of a Myoscape-Conditional KO after in vivo Flp-mediated removal of this selection marker and a Constitutive KO by Cre-mediated deletion of exons 3-8. The estimated deletion of exons 3-8 should result in loss of function by deletion of the N1221 like protein domain and generation of a frameshift
(b). Vector Construction ET: Mouse genomic fragments were ET subcloned using RP23 BAC library and ecloned into the basic targeting vector harbouring the indicated features. If necessary additional fragments were amplified by PCR and subcloned.

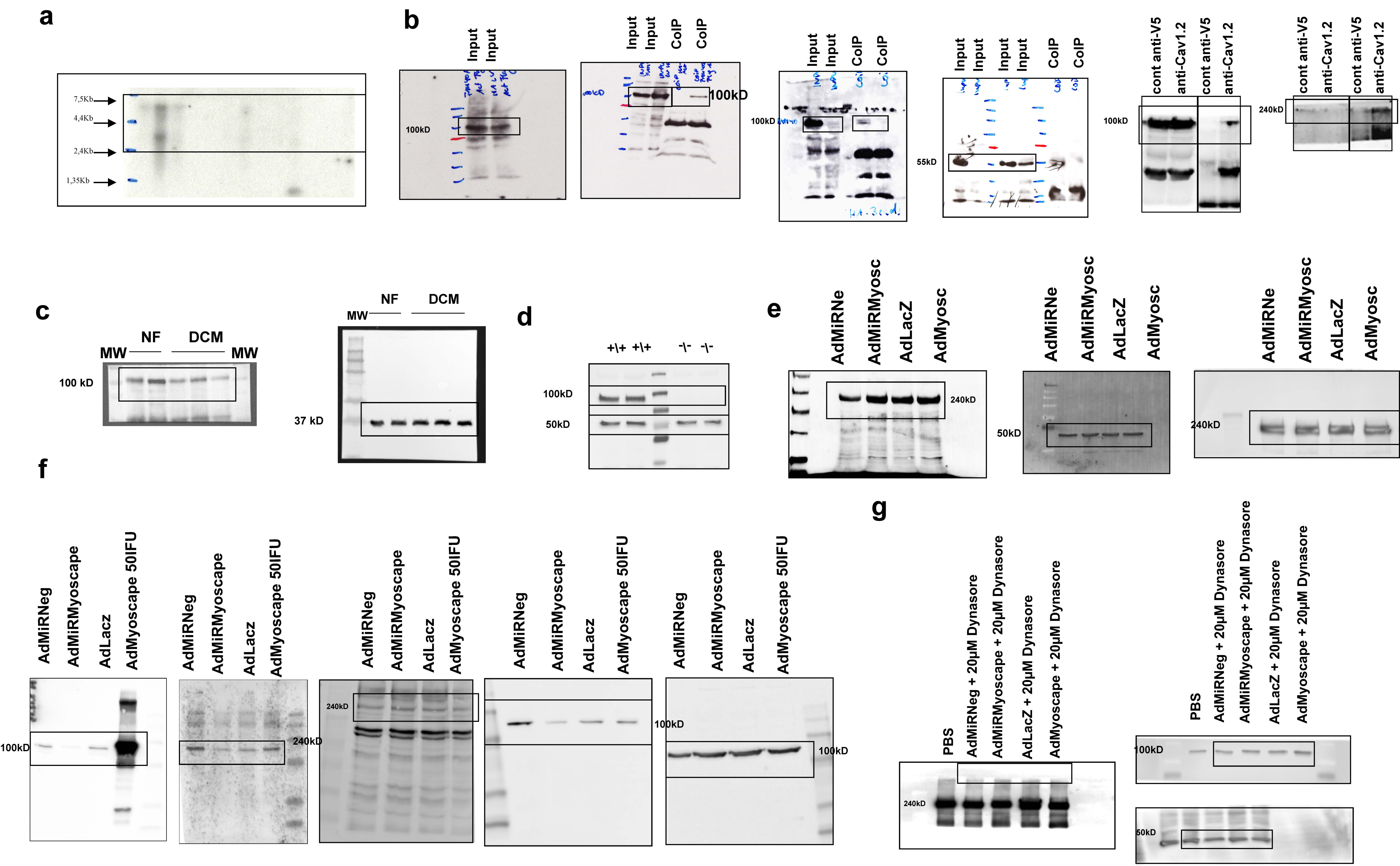

Supplementary Figure 12

Uncropped Blots from Figure 1b (a) and d-f (b), Figure 3f (c), Figure 4b (d) and Figure 5 f,g,h (f and g)

Genotyping protocol of Myoscape Ko mice

PCR ID	Reagents	Primer sequences	PCR protocol	Fragment sizes
1586	5µl PCR Buffer 10x (Invitrogen)<br>2µl MgCl2 (50mM)<br>1µl dNTPs (10mM)<br>1µl Primer 1586_35 (5µM)<br>1µl Primer 1586_36 (5µM)<br>0,4µl Taq (5U/µl, Invitrogen)<br>37,6µl H2O<br>2µl DNA	1586_35:<br>GCAAGGGAATTGATAGACTAGC<br>1586_36:<br>TCCCAGCACCCATATTGTGG	95-C 5'<br>95-C 30''<br>60-C 30''<br>72-C 1'<br>35 cycles<br>72-C 10'	319(wt),<br>438(cond),<br>319(wt)+438(cond)
1587	5µl PCR Buffer 10x (Invitrogen)<br>2µl MgCl2 (50mM)<br>1µl dNTPs (10mM)<br>1µl Primer 1587_39 (5µM)<br>1µl Primer 1587_40 (5µM)<br>0,4µl Taq (5U/µl, Invitrogen)<br>37,6µl H2O<br>2µl DNA	1587_39:<br>GCATTCGGCAATTCTGTACC<br>1587_40:<br>CCATATGCCACCATCCTTGG	95-C 5'<br>95-C 30''<br>60-C 30''<br>72-C 1'<br>35 cycles<br>72-C 10'	315(wt),<br>508(cond),<br>315(wt)+508(cond)
1588	5µl PCR Buffer 10x (Invitrogen)<br>2µl MgCl2 (50mM)<br>1µl dNTPs (10mM)<br>1µl Primer 1586_35 (5µM)<br>1µl Primer 1587_40 (5µM)<br>1µl Primer 1260_1 (5µM)<br>1µl Primer 1260_2 (5µM)<br>0,4µl Taq (5U/µl, Invitrogen)<br>35,6µl H2O<br>2µl DNA	1586_35:<br>GCAAGGGAATTGATAGACTAGC<br>1587_40:<br>CCATATGCCACCATCCTTGG<br>1260_1:<br>GAGACTCTGGCTACTCATCC<br>1260_2:<br>CCTTCAGCAAGAGCTGGGGAC	95-C 5'<br>95-C 30''<br>60-C 30''<br>72-C 1'<br>35 cycles<br>72-C 10'	409(conv)<br>585(control)

Supplementary Table 1:
PCR protocols, reagents and primer sequences in order to detect homozygous/heterozygous wt and conditional alleles in WT and Myoscape KO mice
